# Accessory ESCRT‐III proteins are conserved and selective regulators of Rab11a‐exosome formation

**DOI:** 10.1002/jev2.12311

**Published:** 2023-03-05

**Authors:** Pauline P. Marie, Shih‐Jung Fan, John Mason, Adam Wells, Cláudia C. Mendes, S. Mark Wainwright, Sheherezade Scott, Roman Fischer, Adrian L. Harris, Clive Wilson, Deborah C. I. Goberdhan

**Affiliations:** ^1^ Department of Physiology Anatomy and Genetics University of Oxford Oxford UK; ^2^ Target Discovery Institute University of Oxford Oxford UK; ^3^ Department of Oncology University of Oxford Oxford UK

**Keywords:** ESCRT, CHMP5, extracellular vesicle, Rab11a‐exosome, recycling endosome

## Abstract

Exosomes are secreted nanovesicles with potent signalling activity that are initially formed as intraluminal vesicles (ILVs) in late Rab7‐positive multivesicular endosomes, and also in recycling Rab11a‐positive endosomes, particularly under some forms of nutrient stress. The core proteins of the Endosomal Sorting Complex Required for Transport (ESCRT) participate in exosome biogenesis and ILV‐mediated destruction of ubiquitinylated cargos. Accessory ESCRT‐III components have reported roles in ESCRT‐III‐mediated vesicle scission, but their precise functions are poorly defined. They frequently only appear essential under stress. Comparative proteomics analysis of human small extracellular vesicles revealed that accessory ESCRT‐III proteins, CHMP1A, CHMP1B, CHMP5 and IST1, are increased in Rab11a‐enriched exosome preparations. We show that these proteins are required to form ILVs in *Drosophila* secondary cell recycling endosomes, but unlike core ESCRTs, they are not involved in degradation of ubiquitinylated proteins in late endosomes. Furthermore, *CHMP5* knockdown in human HCT116 colorectal cancer cells selectively inhibits Rab11a‐exosome production. *Accessory ESCRT‐III* knockdown suppresses seminal fluid‐mediated reproductive signalling by secondary cells and the growth‐promoting activity of Rab11a‐exosome‐containing EVs from HCT116 cells. We conclude that accessory ESCRT‐III components have a specific, ubiquitin‐independent role in Rab11a‐exosome generation, a mechanism that might be targeted to selectively block pro‐tumorigenic activities of these vesicles in cancer.

## Synopsis

Analysis in complementary fly and human models shows accessory ESCRT‐III components selectively regulate Rab11(a)‐exosome production in endosomes labelled by recycling endosomal marker Rab11(a). Furthermore, blocking this class of ESCRTs suppresses both pathological and physiological functions associated with exosomes.

- Levels of accessory‐ESCRT‐III proteins are increased in small extracellular vesicle (sEV) preparations enriched with Rab11a‐exosomes; which are preferentially released following glutamine‐depletion of human cancer cell lines.
- Accessory ESCRT‐III proteins are required for Rab11(a)‐exosome biogenesis in recycling endosomes, but not for ESCRT‐dependent processing of ubiquitinylated, late endosomal intraluminal vesicle cargos.
- *Accessory ESCRT‐III* knockdown has a much milder effect on the cellular endolysosomal network than *core ESCRT* knockdown.
- Knockdown of accessory ESCRT‐III proteins suppresses Rab11(a)‐exosome‐mediated human cancer cell growth and post‐mating responses in female flies.

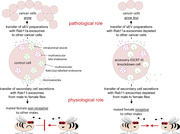

## 1 INTRODUCTION

Exosomes are small extracellular vesicles (sEVs) initially generated as intraluminal vesicles (ILVs) in endosomal multivesicular endosomes (MVEs) (Colombo et al., 2014; Möbius et al., 2002; White et al., 2006). They are released by all cell types and mediate cell‐cell communication events in development, immunity, reproduction and many other physiological processes, as well as diseases such as neurodegenerative disorders (Thompson et al., 2016) and cancer (Saber et al., 2020). For example, exosome cargos can modify the behaviour of recipient cells, leading to tumour progression by driving cancer cell growth and invasiveness, reprogramming the tumour microenvironment, promoting endothelial network assembly, modulating the immune response, and inducing pre‐metastatic niche formation (Becker et al., 2016; Comito et al., 2020; Saber et al., 2020). Exosomes are highly heterogeneous, but the mechanisms underlying this diversity and the routes by which exosome signalling can be modulated have, until recently, remained largely unexplored.

MVEs are widely assumed to be of late endosomal origin. At least two exosome biogenesis mechanisms have been reported in these compartments. One involves the Endosomal Sorting Complex Required for Transport (ESCRT) proteins, a modular ILV‐generation system originally discovered in yeast (Raymond et al., 1992). A second ESCRT‐independent mechanism requires ceramide (Trajkovic et al., 2008). The ESCRT machinery is composed of four protein subcomplexes named ESCRT‐0, ‐I, ‐II, and –III, and the ATPase Vps4. These assemble sequentially at the limiting membrane of the late endosome (Schuh & Audhya, 2014; Vietri et al., 2019). The ESCRT machinery regulates various cellular processes involving membrane remodelling (Henne et al., 2011), including development (Irion & St Johnston, 2007), neurogenesis (Loncle et al., 2015), virus budding (Carlton et al., 2008), cytokinesis (König et al., 2017; Matias et al., 2015) and autophagy (Lee et al., 2007; Rusten et al., 2007). ESCRT malfunction is also associated with numerous human diseases (reviewed in Saksena & Emr, 2009).

ESCRT‐III proteins are subdivided into core and accessory molecules. While the functions of the accessory‐ESCRT‐IIIs, CHMP1, IST1, Vta1 and CHMP5 is unclear in metazoans, they appear to be required for the proper functioning of the ESCRT subcomplex Vps4 in yeast endosomes. CHMP1 associates with Ist1, and Vta1 with CHMP5 in two distinct sub‐complexes (Nickerson et al., 2010). Single *accessory ESCRT‐III* knockdowns or mutants in different species often produce little if any phenotype (Agromayor et al., 2009; Dimaano et al., 2008; Rue et al., 2007), whereas *Vps4* nulls produce severe phenotypes (Babst et al., 1998), impairing ESCRT complex dissociation. Some combinations of *accessory ESCRT‐III* mutations lead to formation of vesicle‐free endosomes in yeast (Nickerson et al., 2010) and *Drosophila* (Bäumers et al., 2019), mimicking *Vps4* knockouts. These findings have led to the suggestion that accessory ESCRT‐III proteins act as acceptors, modulators and/or enhancers of ESCRT‐III‐mediated vesicle scission (Azmi et al., 2008; Frankel et al., 2017; Rue et al., 2007) and may often be individually dispensable. An alternative hypothesis is that they provide robustness to the ESCRT pathway under stress conditions, for example, when cells are highly metabolically active (Agromayor et al., 2009; Loncle et al., 2015; Shim et al., 2006), or at low temperatures (Bäumers et al., 2019).

We have recently shown in a range of human cancer cell lines and in the prostate‐like secondary cells (SCs) of the male accessory gland in the fruit fly, *Drosophila melanogaster*, that exosomes are not only generated in late endosomes, but also in compartments marked by the recycling endosomal trafficking regulator Rab11a (or Rab11 in flies) (Fan et al., 2020). These latter exosomes, some of which are marked by Rab11(a), have different cargos and functions to late endosomal exosomes. In cancer cells, Rab11a‐exosome secretion is induced by metabolic stresses via suppression of nutrient‐sensitive mechanistic Target of Rapamycin Complex 1 (mTORC1) signalling, while exosome secretion from late endosomes is reduced, at least partly via a switch in endosomal trafficking.

Informed by a comparative proteomics analysis of human sEVs enriched for either Rab11a‐exosomes or late endosomal exosomes, we show that the accessory ESCRT‐III genes are essential for the normal biogenesis of Rab11a‐exosomes. Unlike the core ESCRT components, however, they are not required for the ILV‐associated mechanism that removes ubiquitinylated proteins from the surface of late endosomes. The more selective effects of *accessory ESCRT‐III* knockdown is, however, sufficient to suppress EV‐mediated biological functions in both flies and humans, suggesting that Rab11a‐exosomes play a key role in exosome signalling in both normal physiology and disease.

## 2 MATERIALS AND METHODS

### 2.1 Separation of sEVs by differential ultracentrifugation

Cell culture conditions for isolation of sEVs under glutamine‐replete and ‐depleted regimes were previously determined by Fan et al. (2020). HCT116 cells were grown in serum‐free medium (DMEM/F12) supplemented with 1% ITS (Insulin‐Transferrin‐Selenium; #41400045 Life Technologies) for 24 h. For glutamine depletion experiments, cells were grown for 24 h in DMEM/F12 medium without L‐glutamine (#21331046; Life Technologies) supplemented with 1% ITS and 2.00 mM or 0.15 mM glutamine (Life Technologies). Approximately 8–9 × 10^6^ cells were seeded per 15 cm cell culture plate (typically 10–15 plates used per condition) and allowed to settle for 16–18 h in complete medium before the 24 h EV collection. At the end of the collection period, cells were approximately 90% confluent. The culture medium was centrifuged at 500 × *g* for 10 min at 4°C and 2000 × *g* for another 10 min at 4°C to remove cells, debris and large vesicles. The supernatant was filtered to remove larger EVs using 0.22 μm filters (Mile).

Large volumes of the filtrate were concentrated using a tangential flow filtration (TFF) unit with a 100 kDa membrane (Vivaflow 50R, Sartorius) using a 230 V pump (Masterflex). The EV suspension was centrifuged at 108,000 × *g* for 70 min in a Beckman 70 Ti rotor at 4°C (Beckman Coulter). The final EV pellet was resuspended in 70–100 μL PBS for subsequent experiments. For activity, Proteinase K and most NTA assays, EVs were used within 24 h, but for western analysis, they were often stored at ‐80°C.

### 2.2 Separation of sEVs by size‐exclusion chromatography

Collection and initial separation of sEVs by size‐exclusion chromatography followed the same protocol as for differential ultracentrifugation, including the concentration using a TFF unit to a volume of approximately 30 mL. The suspension was then further concentrated in 100 kDa Amicon filters by sequential centrifugation at 4000 × *g* for 10 min at 4°C to a final volume of 1 mL. This was injected into a size‐exclusion column (column size 24 cm × 1 cm) containing Sepharose 4B (84 nm pore size) using an AKTA start system (GE Healthcare Life Science) and eluted with PBS, collecting 30 × 1 mL fractions. Fractions corresponding to the initial “EV peak” (typically fractions two to five) were pooled in 100 kDa Amicon tubes to a final volume of approximately 100 μL.

### 2.3 Comparative proteomics analysis of sEV preparations

Five paired samples of conditioned medium (serum‐free basal medium [DMEM/F12] supplemented with 1% ITS [Insulin‐Transferrin‐Selenium; #41400045 Life Technologies]) were collected over a 24‐h period from glutamine‐depleted and glutamine‐replete HCT116 cells, and sEVs isolated by differential ultracentrifugation and resuspended in PBS (Fan et al., 2020).

Samples were lysed in 100 μL RIPA buffer for 30 min on ice, followed by 10 min centrifugation at 17,000 × *g* at 4°C. The clear supernatants were transferred into new tubes. Samples were reduced with DTT (final concentration 5 mM) and alkylated with Iodoacetamide (final concentration 20 mM) for 30 min each and then precipitated with methanol/chloroform. In brief, samples were mixed with 600 μL methanol, 150 μL chloroform and 450 μL ddH2O. After centrifugation for 3 min at 17,000 × *g*, the upper phase was carefully removed and another 450 μL methanol were added. After another centrifugation step for 5 min, supernatants were taken off completely and discarded. The protein pellets were resuspended in 50 μL 100 mM TEAB and digested with 200 ng trypsin (Promega sequencing grade) overnight at 37°C.

Sample amounts were normalized based on protein concentration measurements for TMT labelling. Approximately 5.6 μg of peptides in 50 μL 100 mM TEAB were labelled with 0.06 mg of a TMT10plex label for 1 h and then quenched with 5 μL 400 mM Tris‐HCl for 15 min. All 10 samples were combined, desalted on SOLA HRP SPE cartridges (Thermo Scientific) and dried down in a vacuum centrifuge. For LC‐MS/MS, samples were resuspended in 2 % acetonitrile with 0.1% formic acid.

Samples were analysed on the Dionex Ultimate 3000/Orbitrap Fusion Lumos platform. Peptides were separated on a 50 cm, 75 μm ID EasySpray column (ES803; Thermo Fisher) on a 60‐minute gradient of 2 to 35% acetonitrile (containing 0.1% formic acid and 5% DMSO) at flow rate of 250 nl/min. Data were acquired using the MultiNotch MS3 method as described previously (McAlister et al., 2014).

Mass spectrometry raw data were analysed in Proteome Discoverer 2.1. Proteins were identified with Sequest HT against the UPR Homo sapiens database (retrieved February 2017). Mass tolerances were set to 10 ppm for precursor and 0.5 Da fragment mass tolerance. TMT10plex (N‐term, K), Oxidation (M) and Deamidation (N, Q) were set as dynamic modifications, alkylation (C) as a static modification. The mass spectrometry proteomics data have been deposited to the ProteomeXchange Consortium via the PRIDE (Vizcaíno et al., 2016) partner repository with the dataset identifier PXD040361.

### 2.4 *Drosophila* stocks and genetics

The following fly stocks were acquired from the Bloomington *Drosophila* Stock Centre [RNAi lines from the TRiP collection (Ni et al., 2009)] and the Vienna *Drosophila* Research Center [RNAi lines from the shRNA, GD and KK libraries (Dietzl et al., 2007)] were used, unless otherwise stated: *UAS‐rosy*‐RNAi‐#1 (TRiP.HMS02827, BL 44106), *UAS‐Hrs*‐RNAi‐#1 (TRiP.JF02860, BL 28026), ‐#2 (TRiP.HMS00841, BL 33900), *UAS‐Stam*‐RNAi‐#1 (GD 11948, VDRC 22497), ‐#2 (TRiP.HMS01429, BL 35016), ‐#3 (shRNA 330248, VDRC 330248), *UAS‐TSG101*‐RNAi‐#1 (TRiP.GLV21075, BL 35710), ‐#2 (GD 14295, VDRC 23944), *UAS‐Vps28*‐RNAi‐#1 (GD 7696, VDRC 31894), ‐#2 (KK 101474, VDRC 105124), *UAS‐Vps36*‐RNAi‐#1 (TRiP.HMS01739, BL 38286), ‐#2 (KK 102099, VDRC 107417), *UAS‐Vps25*‐RNAi‐#1 (GD 8432, VDRC 38821), ‐#2 (KK 102944, VDRC 108105), ‐#3 (TRiP.JF02055, BL 26286), *UAS‐shrb*‐RNAi‐#1 (KK 108557, VDRC 106823), ‐#2 (TRiP.HMS01767, BL 38305), *UAS‐Vps24*‐RNAi‐#1 (GD 14676, VDRC 29275), ‐#2 (TRiP.HMS01733, BL 38281), ‐#3 (KK 107601, VDRC 100295), *UAS‐Vps20*‐RNAi‐#1 (GD 11211, VDRC 26387), ‐#2 (discontinued, VDRC 47653), *UAS‐* *chmp2*‐RNAi‐#1 (GD 8363, VDRC 24869), ‐#2 (TRiP.HMS01911, BL 38995), *UAS‐Chmp1*‐RNAi‐#1 (GD 11219, VDRC 21788), ‐#2 (TRiP.HM05117, BL 28906), *UAS‐Ist1*‐RNAi‐#1 (KK 108546, VDRC 100771), #2 (GD 6866, VDRC 31174), *UAS‐Chmp5*‐RNAi‐#1 (KK 109120, VDRC 101422), ‐#2 (GD 10565, VDRC 25990) *UAS‐Vps4*‐RNAi‐#1 (KK 101722, VDRC 105977), ‐#2 (GD 12054, VDRC 35125). Most of these lines have been validated by previous independent screens (Bras et al., 2012; Gomez‐Lamarca et al., 2015; Loncle et al., 2015; Mamińska et al., 2016; Neyen et al., 2016; Sheng et al., 2016). The *w; TI{TI}Rab11EYFP* line was a gift from Suzanne Eaton (Dunst et al., 2015), esgF/Ots [*esg‐GAL4 tub‐GAL80ts UAS‐FLP/CyO; UAS‐GFPnls actin > FRT > CD2 > FRT > GAL4/TM6*] was from Bruce Edgar (Jiang et al., 2009), *UAS‐Dad* (Tsuneizumi et al., 1997) was from Daimark Bennett, and the *dsx‐GAL4* line was from Stephen Goodwin (Rideout et al., 2010). The *UAS‐Btl‐GFP* line was obtained from the BDSC (BL 41802), and the *UAS‐GFP‐Hrs* was produced by SJF with Ben Kroeger.

Flies were grown at 25°C in vials containing standard cornmeal agar medium (per litre, 12.5 g agar, 75 g cornmeal, 93 g glucose, 31.5 g inactivated yeast, 8.6 g potassium sodium tartrate, 0.7 g calcium, and 2.5 g Nipagen dissolved in 12 mL ethanol). They were transferred onto fresh food every 3–4 days. No additional dried yeast was added to the vials. The driver line *dsx‐GAL4* and the temperature‐sensitive, ubiquitously expressed, repressor *tubulin‐GAL80ts* were used to induce SC‐specific expression of *UAS‐Btl‐GFP*, as well as RNAis, in a temperature‐controlled fashion. Expression of the *YFP‐Rab11* gene trap was controlled by Rab11 regulatory sequences. Females carrying *dsx‐GAL4, tubulin‐GAL80ts* and one of the three compartment markers were crossed with males carrying each UAS‐RNAi transgene. Virgin male offspring, isolated after eclosion, were transferred to 29°C for 6 days to induce post‐differentiation SC‐specific expression of inducible markers and RNAi.

### 2.5 Preparation of accessory glands for live imaging

Accessory glands were prepared following the protocol described by Fan et al., 2020.

### 2.6 Microscopy

Live imaging was performed at room temperature following the method described by Fan et al. (2020) using the inverted wide‐field fluorescence system DeltaVision Core from Olympus AppliedPrecision (GE Healthcare Life Sciences) equipped with a 100×/1.4 UPlanSApo oil objective (Olympus) with an EM‐CCD camera. Immersion oil RI 1.514 (Zeiss) was used. Images and z‐sections for quantification of intracellular compartments and secretion were captured with 0.3 μm spacing using the SoftWoRx 5.5 software package (GE Healthcare Life Sciences) followed by deconvolution using the Resolve 3D‐constrained iterative deconvolution algorithm. Fixed tissues were imaged with an upright laser scanning confocal Zeiss LSM880. A Zeiss Plan Apochromat oil differential interference contrast objective 63×, NA 1.4 Plan APO oil DIC objective (Carl Zeiss) was used with RI 1.514 immersion oil (Zeiss). Z‐sections and images for quantification of intracellular compartments were captured with 0.5 μm spacing using the Zen Blue suite software according to previously published protocols (Corrigan et al., 2014). As previously reported (Prince et al., 2019; Fan et al., 2020), fixation disrupts SC subcellular morphology, when compared to imaging of living *ex vivo* glands.

### 2.7 Secondary cell compartment analysis

Compartments within living SCs from 6‐day‐old *Btl‐GFP*‐ or *YFP‐Rab11*‐expressing flies (with or without RNAi expression) were analysed. The total number of compartments was quantified. Non‐acidic compartments and ILV‐containing compartments were counted following the methods described in Fan et al. (2020), except all compartments with diameter ≥0.4 μm were included. Three different SCs were analysed from each of 10 glands. Acidic compartments were quantified and measured using the “wand” tool in Fiji software. Dense cores were detected by DIC. If the DCG in a compartment was fragmented, smaller in relation to its compartment than control or absent, the compartment was scored as abnormal. For each SC, the percentage of normal DCG compartments was quantified.

### 2.8 Analysis of ubiquitin accumulation in SCs

Glands were dissected from virgin 6‐day‐old controls and males expressing *ESCRT*‐RNAis in their SCs. They were fixed in 4% paraformaldehyde dissolved in PBS for 15 min to preserve tissue integrity and washed in PBST (PBS and 0.3% Triton X‐100) for 5 min. Glands were pre‐incubated with 10% goat serum in PBST for 1 h and then incubated overnight with primary antibody against GFP (rabbit monoclonal; Abcam #183734, 1:500) and conjugated ubiquitin (Ubi FK2 mouse monoclonal #04‐263; Millipore, 1:500) in PBST. Glands were rinsed five times for 10 min in PBST and incubated overnight in PBST with secondary antibody (Alexa555 anti‐mouse, 1:400 and Alexa448 anti‐rabbit, 1:400; Abcam), then rinsed five more times with PBS. Finally, glands were mounted on a glass slide and immersed in a drop of Vectashield with DAPI (Vector Laboratories) and covered with a coverslip for imaging by confocal microscopy.

### 2.9 *Drosophila* exosome secretion assay

Virgin 6‐day‐old SC > Btl‐GFP‐expressing males were dissected in ice‐cold PBS. Exosome secretion was measured by acquiring a 10 μm z stack within the central third of each gland, with each image spaced by 0.3 μm, using the DeltaVision Elite wide‐field microscope for the *UAS‐Btl‐GFP* marker. Fluorescence coming from nearby SCs interferes with the acquisition in wide‐field microscopy, so the distal tip was not imaged. Images were post‐processed by deconvolution as described in the Microscopy section. The automated analysis of exosome secretions by SCs was performed using ImageJ2 (Schindelin et al., 2015), distributed by Fiji. First the stack was reduced to one image by z‐projection, the central upper area of the image was cropped to avoid edge distortion. A “blank” image was acquired in a gland not expressing any marker and subtracted from the image of the gland to reduce auto fluorescence noise. A threshold was set for each lumenal area using the Triangle method (Zack et al., 1977) to determine the outlines of bright fluorescent particles. The ‘Analyse Particles’ function was then used to determine the total number of fluorescent puncta between 0.1 and 8 μm^2^.

### 2.10 *Drosophila* receptivity assay

Virgin male flies carrying the *esgF/O^ts^* transgene combination (Jiang et al., 2009), which permits SC‐specific, UAS‐mediated gene expression when induced in adults by temperature shift to 29°C, and appropriate knockdown or overexpression constructs (for *Chmp5*, RNAi line #2), were collected at eclosion and cultured at 29°C for 6 days. They were then separated and each was placed into a vial containing a single virgin female of the *w* ^*1118*^ genotype. These vials were incubated at 29°C for 24 h to permit mating, then the females separated into individual vials, whilst the males were placed back at 29°C for a further 24 h with a new *w* ^*1118*^ virgin female. This was repeated one more time so that in total males were incubated with three virgin females for three separate 24‐h periods.

After being separated from the males, female flies were kept in individual vials at 19°C for 3 days. On the third day, females were flipped into new vials. The old vials were retained to check larvae had been produced, thereby confirming that mating had occurred during the initial 24‐h incubation with the *esgF/O^ts^* males.

Once in the new vials, individual Canton S males that were 4–8 days old were added to the individual females. The vials were then observed for 1 h at room temperature and without disturbance to see whether females remated. The males and females were separated (without anaesthesia) and the females retained in individual vials at 19°C. For females that did not remate, the remating assay was repeated for 1 h every day at approximately the same time each day. For any females that died or escaped before they remated, the date of their loss was recorded.

### 2.11 Inducible *Chmp5* knockdown in human HCT116 cells

Using methods previously described by Fan et al. (2016), stably transduced HCT116 clones carrying IPTG‐inducible lentiviral constructs that express a non‐targeting (shNT, CAACAAGATGAAGAGCACCAA, SHC 202, Sigma) or *Chmp5*‐targeting (shCHMP5, CTGGATGAAGATGATTTAGAA, Sigma, TRCN0000158654) shRNA were generated. To eliminate non‐transduced cells, puromycin treatment was performed 2 days after transduction. Single‐cell clones were isolated using the limiting dilution method. qRT‐PCR was performed to screen for clones with strong *Chmp5* mRNA knockdown after IPTG induction.

Following generation of clones, IPTG (100 μM, Sigma) was used to induce shRNA transcription. After three days, cells were plated at a density of nine million cells in a 15 cm cell culture plate in full basal media (2 mM glutamine), incubated overnight until they reached 80% confluency and the media exchanged for sEV‐isolation media (glutamine‐depleted [0.15 mM glutamine] DMEM/F12). Cells were incubated overnight and the following day sEVs were isolated by SEC and analysed, as previously described (Fan et al., 2020). sEVs were quantified using NTA and characterised by western blot (see below), following MISEV2018 guidelines (Théry et al., 2018), before testing their functional effect on proliferation of HCT116 cells plated at a density of 2000 cells per well in 96‐well plates with eight technical repeats per experimental condition using an sEV dose of 4000 sEVs per cell. Proliferation was measured using the IncuCyte ZOOM Live Cell Imager, taking four phase‐contrast images per well every 6 h for 5 days. The IncuCyte analysis software was used to detect cell edges to create a confluency mask, which was used to determine cell growth.

### 2.12 Western analysis

Both cell lysates and EV preparations, which were lysed in RIPA or 1X sample buffer, were electrophoretically separated using 10% mini‐PROTEAN precast gels (BioRad). Gels were loaded for western analysis with EV lysates extracted from the same protein mass of secreting cells, so that changes in band intensity on the blots with glutamine depletion reflected a net change in secretion of the marker on a per cell basis (see Fan et al., 2020). Protein preparations were ultimately dissolved in either reducing (containing 5% β‐mercaptoethanol) or non‐reducing (for CD63 and CD81 detection) sample buffer and were heated to 90°C–100°C for 10 min before loading with a pre‐stained protein ladder (Bio‐Rad). Proteins were wet‐transferred to polyvinylidene difluoride (PVDF) membranes at 100 V for 1 h using a Mini Trans‐Blot Cell (Bio‐Rad). Membranes were then blocked with either 5% milk (CD63 detection) or 5% BSA in TBS buffer with Tween‐20 (TBST) for 30 min and probed overnight at 4°C with primary antibody diluted in blocking buffer. The membranes were washed for 3 × 10 min with TBST, then probed with the relevant secondary antibodies for 1 h at 22°C, washed for 3 × 10 min again, and then the signals detected using the enhanced chemiluminescent detection reagent (Clarity, BioRad) and the Touch Imaging System (BioRad). Relative band intensities were quantified by ChemiDoc software (Bio‐Rad) or ImageJ. Signals were normalised to cell lysate protein (Fan et al., 2020).

Antibody suppliers, catalogue numbers and concentrations used were: rabbit anti‐CHMP1a (Proteintech #15761‐1‐AP, 1:500), rabbit anti‐CHMP1b (Proteintech #14639‐1‐AP, 1:500), rabbit anti‐IST1 (Biorad #VPA00314, 1:500), mouse anti‐CHMP5 (Santa Cruz #sc‐374338, 1:500), rabbit anti‐4E‐BP1 (Cell Signaling Technology #9644, rabbit anti‐p‐4E‐BP1‐Ser65 (Cell Signaling Technology #9456, 1:1000), rabbit anti‐S6 (Cell Signaling Technology #2217, 1:4000), rabbit anti‐p‐S6‐Ser240/244 S6 (Cell Signalling Technology #5364, 1:4000), rabbit anti‐Caveolin‐1 (Cell Signaling Technology #3238, 1:500), goat anti‐AREG (R&D Systems #AF262, 1:200), mouse anti‐Tubulin (Sigma #T8328, 1:4000), mouse anti‐CD81 (Santa Cruz #23962, 1:500), mouse anti‐CD63 (BD Biosciences # 556019, 1:500), rabbit anti‐Syntenin‐1 antibody (Abcam ab133267, 1:500), rabbit anti‐Tsg101 (Abcam ab125011, 1:500), mouse anti‐Rab11 (BD Biosciences #610657, 1:500), sheep anti‐TGN46 (BioRad; AHP500G, 1:1000), rabbit anti‐EEA1 (Cell Signalling Technology #3288, 1:1000) , anti‐mouse IgG (H+L) HRP conjugate (Promega #W4021, 1:10000), anti‐rabbit IgG (H+L) HRP conjugate (Promega #W4011, 1:10000), anti‐goat IgG (H+L) HRP conjugate (R&D Systems #HAF109, 1:100).

### 2.13 Statistical analysis

In all analyses, mean values per secondary cell are presented for pooled data and error bars are SD. For comparison between control and RNAi lines, statistical significance was determined using multiple comparisons with one‐way ANOVA test for parametric data. Most data for compartment number counts, and percentage of compartments producing ILVs or DCGs failed the Shapiro‐Wilk normality test (*p* < 0.01). As a result, the non‐parametric Kruskal‐Wallis test was used to test significance of differences under all knockdown conditions. Statistical analyses were performed using GraphPad Prism version 8. 3. 1. *p < 0.05, **p < 0.01, ***p < 0.001 and ****p < 0.0001 relative to control. For the Btl‐GFP marker, *Control #1* was used for the multiple comparison with RNAi lines for ESCRT‐0‐I‐II and *shrb*, and *Control #2* with RNAi lines for core and accessory ESCRT‐III, as well as *Vps4*. Therefore, *Ctrl* in Figure 2f‐h is the same as in Figure S3f‐h. The remaining data were analysed using a Gehan‐Breslow‐Wilcoxon test. For Western blots, relative signal intensities were compared using the Kruskal‐Wallis test. For the growth assays, data were analysed by one‐way ANOVA.

## 3 RESULTS

### 3.1 Accessory ESCRT‐III proteins are increased in sEV preparations enriched in Rab11a‐exosomes

**FIGURE 1:**
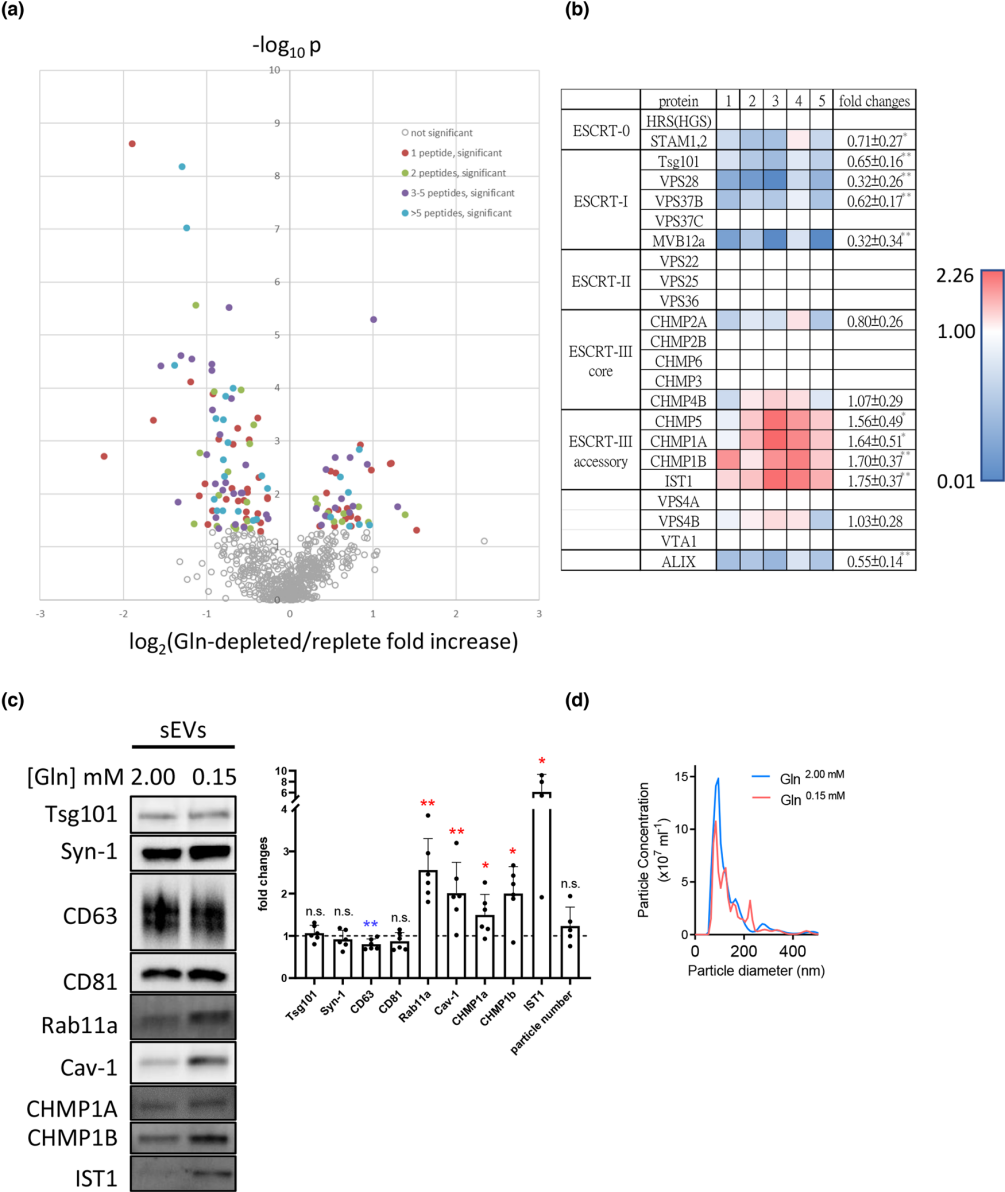
sEV preparations from glutamine‐depleted HCT116 colorectal cancer cells contain elevated levels of accessory ESCRT‐III proteins. Five paired protein samples made from sEVs isolated by differential ultracentrifugation from glutamine‐depleted and glutamine‐replete HCT116 cells were subjected to TMT‐labelled comparative proteomics analysis. (a) Volcano plot showing mean log_2_ fold change in specific protein levels in sEV preparations from glutamine‐depleted conditions versus glutamine‐replete conditions plotted against ‐log_10_ *p* value for null hypothesis that levels are unchanged. Coloured data points represent proteins that are significantly altered in levels between the two conditions, with colours representing number of peptides detected. (b) Heat map showing change in levels of core and accessory ESCRT proteins in five paired sEV samples (labelled 1 to 5) from glutamine‐depleted versus glutamine‐replete HCT116 cells. Red shows an increase in protein levels and blue indicates a reduction following glutamine depletion. (c) Western blot of sEV preparations confirms that accessory ESCRT‐III proteins CHMP1A, CHMP1B and IST1 are all increased in sEVs produced by glutamine‐depleted (0.15 mM) HCT116 cells. Bar chart shows protein levels and sEV number normalised to cell lysate. Data derived from five independent experiments and analysed by the Kruskal‐Wallis test. Significantly changed levels are denoted by a blue (decreased) and red (increased) asterisks. *p < 0.05; n.s. = not significant. (d) Nanosight Tracking Analysis of sEV size and number for samples produced as in (c)

To further investigate the specific properties of Rab11a‐exosomes, we isolated five paired preparations of sEVs from HCT116 colorectal cancer cells under glutamine‐depleted and glutamine‐replete conditions. Reduced glutamine levels suppress signalling by the nutrient‐sensitive mTORC1 kinase complex, which induces elevated secretion of Rab11a‐exosomes without having a significant effect on cell survival (Fan et al., 2020) or overall secreted sEV number, as determined by Nanosight analysis of particle counts (Figure 1c,d). We used high‐throughput Tandem‐Mass Tag (TMT) mass spectrometry to compare the proteomes of the paired sEV preparations.

A volcano plot of the resulting data (Figure 1a) revealed that of the 683 proteins identified in all 10 samples, 48 were significantly increased in Rab11a‐exosome‐enriched sEV preparations (right side of y‐axis in Figure 1a; Table S1) and 87 were significantly decreased (left side of y‐axis in Figure 1a; Table S2). An analysis of enriched GO terms within the 683 proteins revealed that many were associated with exosomes, endosomal microautophagy, viral budding and release, plus ESCRT biology (Table S3), confirming the vesicular nature of these sEV preparations. Intriguingly, the most enriched GO terms in the significantly increased proteins were ESCRT‐III complex disassembly and ESCRT complex disassembly (Table S4). The accessory ESCRT‐III proteins CHMP1A, CHMP1B, CHMP5 and IST1 were the only proteins in this group (Figure 1b). By comparison, members of most classes of core ESCRTs were not consistently increased or reduced in sEVs produced under nutrient‐depleted conditions with the exception of the ESCRT‐I protein family, where most members appeared to be reduced in Rab11a‐exosome‐enriched sEV preparations (Figure 1b). However, for the ESCRT‐I, Tsg101, the apparent small reduction seen by proteomics was not confirmed by western blot (Figure 1c).

In sharp contrast to these findings, positive regulation of ubiquitin‐dependent endocytosis and regulation of ubiquitin‐dependent endocytosis were among the most enriched terms in sEV preparations from glutamine‐replete cells (Table S5). Terms associated with ubiquitin‐dependent catabolism in multivesicular bodies were also highly enriched specifically in this protein group.

Although the levels of CHMP5 protein in sEVs were too low to detect by western analysis, this approach was used to confirm elevated levels of the accessory ESCRT‐III proteins CHMP1A, CHMP1B and IST1 in sEVs collected from glutamine‐depleted cells (Figure 1c,d); levels of these proteins in cell lysates were either unaffected or reduced by this treatment (Figure S1a). Unlike the membrane‐associated tetraspanins, CD63 and CD81, these proteins were not concentrated in exosomes (Figure S1b), but similar to Rab11a, their overall levels in sEVs were increased following glutamine‐depletion (Figure 1c).

The accessory ESCRT‐III proteins have been reported to be closely associated with the cytosolic side of the cellular lipid bilayer and should therefore be recruited to the inside of EVs (McCullough et al., 2015). To confirm that these proteins were not present as soluble contaminants in sEV preparations from glutamine‐depleted cells, we investigated whether CHMP1B, like other proteins on the inside of EVs, such as Syn‐1 and Tsg101, was resistant to digestion by Proteinase K (Figure S1c). CHMP1B was not digested by Proteinase K. Indeed, even in the presence of low levels of the detergent Triton‐X100, which partially disrupts the lipid bilayer and permits digestion of Syn‐1 and Tsg101, full‐length CHMP1B protein persisted, perhaps because of its close association with lipids. Taken together, these findings suggested differences in the ESCRT‐mediated mechanisms controlling Rab11a‐exosome and late endosomal exosome biogenesis, which we investigated further, initially in *Drosophila* SCs.

### 3.2 Core ESCRT proteins are required for ILV biogenesis in Rab11‐positive compartments of *Drosophila* secondary cells

**FIGURE 2:**
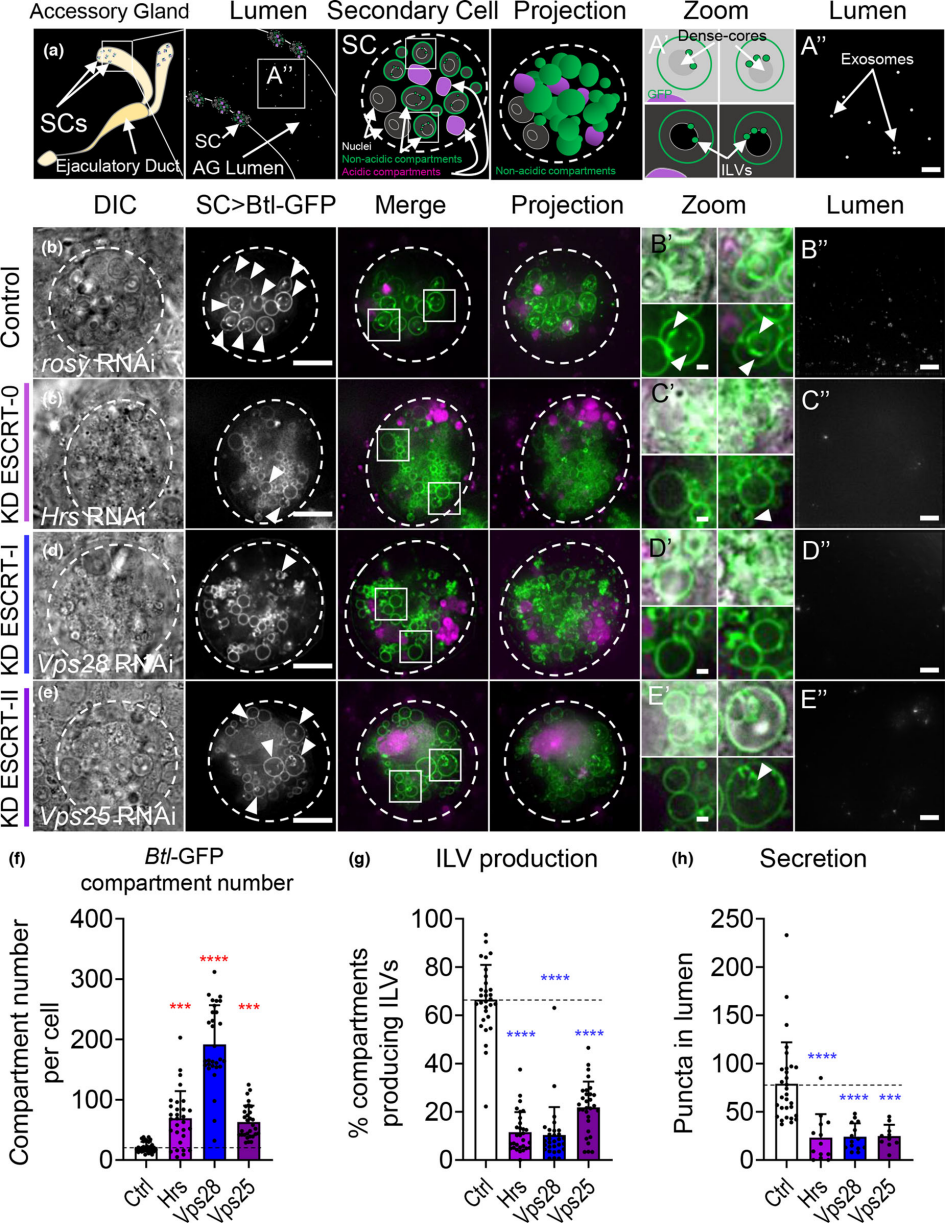
ESCRT‐0, ‐I and ‐II components regulate exosome biogenesis in non‐acidic compartments of *Drosophila* SCs. (a) Schematic of accessory gland (AG), marking position of secondary cells (SCs) at distal tips of both lobes (first panel), transverse section through AG lumen and secondary cells (second panel) and schematic representation of a basal Z‐plane view of a single SC (third panel), which corresponds to the SC images in columns 1–3 (B‐E). This SC schematic highlights large non‐acidic compartments in green (marked by Btl‐GFP), and late endosomes and lysosomes in magenta. Schematic of a Z‐stack projection through the same SC (fourth panel). The outline of each SC in the schematics and images is approximated by a dashed circle. Note that both SCs and main cells are binucleate. As in the images (b‐e), two boxed non‐acidic compartments (third panel) are magnified in separate zoom images (A’). This allows DCGs to be visualised via DIC (top), and without DIC (bottom) highlights ILVs. In some cases (B’), the DCG is completely surrounded by fluorescent puncta, so the DIC outline cannot be easily seen. The central region of the lumen is shown schematically (A’’; 10 μm projection with 0.3 μm spacing) and in images (B’’‐E’’).Panels B‐E (columns 1–3) show basal wide‐field fluorescence views of living SCs from 6‐day‐old males expressing a *UAS‐Btl‐GFP* (green) and a UAS‐RNAi construct under temperature‐induced, SC‐specific GAL4 control from eclosion onwards. Acidic compartments are marked by LysoTracker Red (magenta). (B) SC expressing Btl‐GFP and *rosy*‐RNAi construct (control). Btl‐GFP‐positive ILVs (arrowheads; B’ bottom) are observed inside large non‐acidic compartments (arrowheads in SC > Btl‐GFP) and as puncta in AG lumen (B’’). DCGs are visible in mature Btl‐GFP‐positive compartments (DIC; B’ top). (c) SCs expressing *Hrs*‐RNAi lack DCGs (DIC; first column) and have a reduced proportion of Btl‐GFP‐positive ILV‐containing compartments (c’ bottom, g) and secreted puncta, representing secreted exosomes (c’’, h). (d) SCs expressing *Vps28*‐RNAi also have less DCGs and ILV‐containing compartments (D’ bottom, F), and have reduced exosome secretion (D’’, H). (e) SCs expressing *Vps25*‐RNAi form DCGs and enlarged ILVs in some non‐acidic compartments (E’ bottom; arrowhead). Exosome secretion is reduced (E’’, H). (f) Bar chart showing the number of non‐acidic Btl‐GFP‐positive compartments per SC with diameter > 0.4 μm in control versus ESCRT‐0 (*Hrs*), ‐I (*Vps28*), and ‐II (*Vps25*) knockdowns (*n* = 30 SCs). (g) Bar chart showing percentage of Btl‐GFP compartments containing Btl‐GFP‐positive ILVs for different genotypes (*n* = 30 SCs). (h) Bar chart showing number of Btl‐GFP‐positive fluorescent puncta in the lumen of AGs with ESCRT knockdown compared to control (*n* ≥ 10). Genotypes are: *w; P[w+, tub‐GAL80ts]/+; dsx‐GAL4/P[w+, UAS‐btl‐GFP]/+* with *UAS‐rosy*‐RNAi knockdown construct (b), *UAS‐Hrs*‐RNAi‐#1 (c), *UAS‐Vps28*‐RNAi‐#1 (d), *UAS‐Vps25*‐RNAi‐#1 (e). Scale bars in B‐E and B’’‐E’’, 10 μm; in B’‐E’, 1 μm. Data were analysed by Kruskal‐Wallis test. ***p < 0.001 and ****p < 0.0001 relative to control. See also Figure S3 for additional *ESCRT* knockdowns tested, Figure S4 for second RNAi lines analysed (#2) and YFP‐Rab11 analysis, Figure S5 for representative DCG images

**FIGURE 3:**
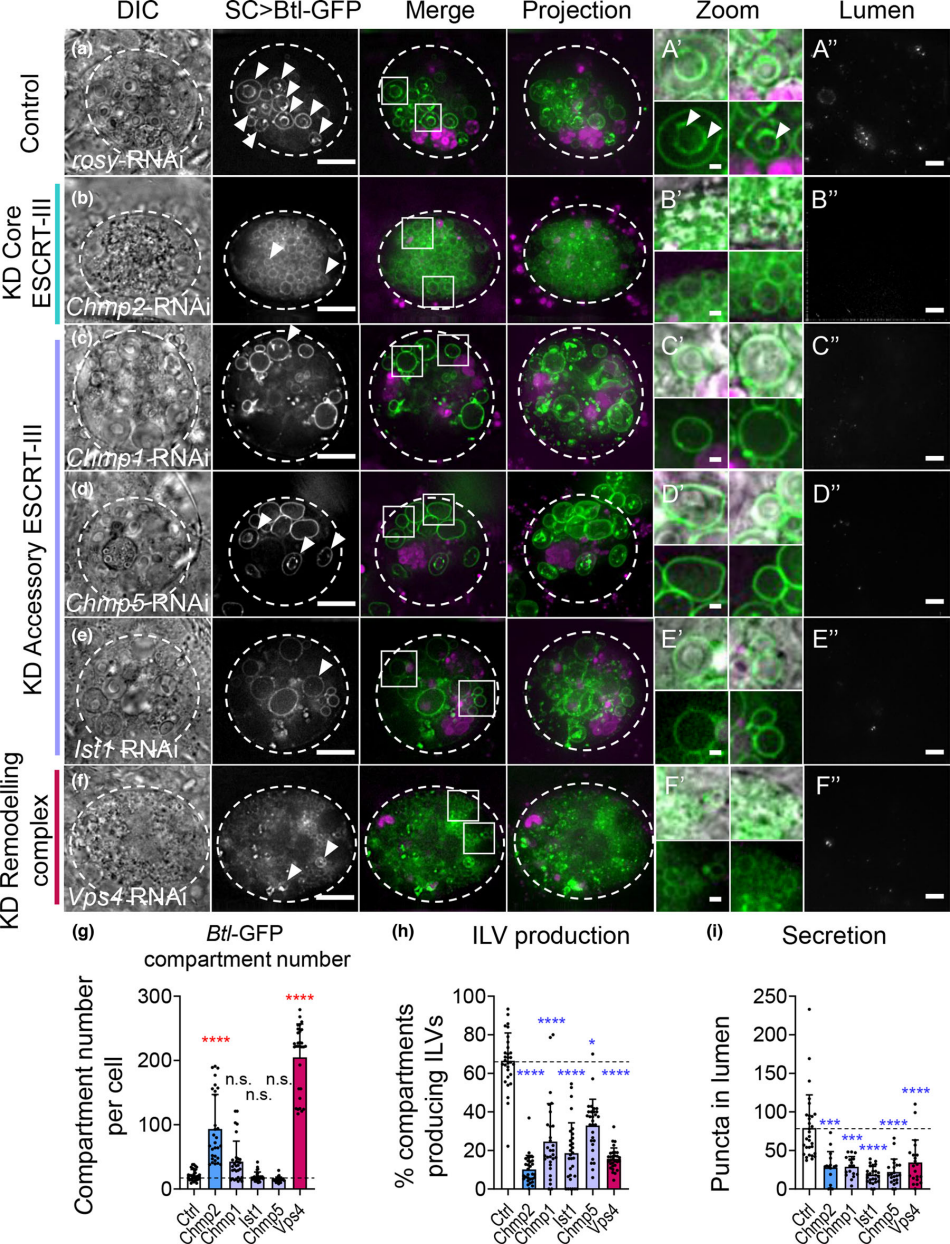
Both core and accessory ESCRT‐III components regulate exosome biogenesis in non‐acidic compartments of SCs. Panels A‐F show basal wide‐field fluorescence views of living SCs expressing Btl‐GFP (green; control schematic, Figure 2a, third panel). SC outline approximated by dashed white circles. Acidic compartments are marked by LysoTracker Red (magenta). Boxed non‐acidic compartments are magnified in A’‐F’ (Zoom). Transverse image projections of AG lumens are shown in A’’‐F’’. (a) Control SC expressing *rosy*‐RNAi. Btl‐GFP‐positive vesicle membranes are observed inside nearly 70% of large non‐acidic compartments (arrowheads; A’) and as puncta in AG lumen (A’’). (b) SC expressing *Chmp2‐*RNAi. Non‐acidic compartment number is increased (g). Btl‐GFP‐positive ILVs (B’ bottom, H) and puncta in the AG lumen are reduced (B’’, I), as are DCGs. Note the large lysosomes in surrounding main cells (B Projection), which are sometimes observed in glands containing *ESCRT*‐knockdown SCs. (c) SC expressing *Chmp1‐*RNAi. In this cell, non‐acidic compartment number is increased (g). Btl‐GFP‐positive ILVs, DCGs and puncta in the AG lumen are reduced (C’’, I). (d) SC expressing *Chmp5*‐RNAi. Btl‐GFP‐positive ILVs and puncta in the AG lumen are reduced (D’’, I). (e) SC expressing *Ist1*‐RNAi. Btl‐GFP‐positive ILVs, DCGs and puncta in the AG lumen are reduced (E’’, I). (f) SC expressing *Vps4*‐RNAi. Btl‐GFP‐positive compartments are greatly increased. Btl‐GFP‐positive ILVs, DCGs and puncta in the AG lumen are reduced (F’’, I). (g) Bar chart showing the number of non‐acidic Btl‐GFP‐positive compartments with diameter > 0.4 μm per SC in control vs *ESCRT‐III* knockdowns. Note that *accessory ESCRT‐III* knockdowns do not significantly affect overall compartment organisation and morphology, unlike *Chmp2* and *Vps4* knockdown. *n* = 30 SCs. (h) Bar chart showing percentage of Btl‐GFP‐positive compartments containing Btl‐GFP‐positive ILVs. *n* = 30 SCs. (i) Bar chart showing Btl‐GFP fluorescent puncta number in the lumen of AGs with *ESCRT‐III* knockdowns compared to controls. *n* ≥ 10 AG lumens.All data are from 6‐day‐old male flies shifted to 29°C at eclosion to induce expression of transgenes. Genotypes are: w; *P[w+, tub‐GAL80^ts^]/+; dsx‐GAL4/P[w+, UAS‐btl‐GFP]/+* with *UAS‐rosy‐RNAi* knockdown construct (a), *UAS‐Chmp2*‐RNAi‐#1 (b), *UAS‐Chmp1‐RNAi*‐#1 (c), *UAS‐Chmp5‐RNAi*‐#1 (d), *UAS‐I*st1‐RNAi‐#1 (e), *UAS‐Vps4*‐RNAi‐#1 (f). Scale bars in A‐F and A’’‐F’’, 10 μm, and in A’‐F’, 1 μm. Data were analysed by Kruskal‐Wallis test. *p < 0.05, ***p < 0.001 and ****p < 0.0001 relative to control. See Figure S4a‐c for additional RNAi lines tested

The *Drosophila* accessory gland is composed of two cell types, the main cells and the secondary cells (SCs; Figure 2a). SCs have unusually large secretory and endosomal compartments, including between three and five large (>2 μm diameter) Rab7‐positive late endosomes and lysosomes (LELs), which can be visualised with LysoTracker staining, and depending on the marker used, between ten and twenty large (>2 μm diameter) non‐acidic compartments can be seen (Corrigan et al., 2014; Fan et al., 2020) (Figure 2a). The latter can be fluorescently marked when a GFP‐tagged form of Breathless (Btl), a fly FGF receptor homologue, is overexpressed in SCs (Figures 2, 3). Btl‐GFP also labels all or most ILVs (Figure 2a’,b’ Zoom) (Fan et al., 2020), collectively called Rab11‐exosomes, with some particularly bright puncta often apparent. These puncta typically surround a central, protein‐rich dense‐core granule (DCG), which can be visualised with differential interference contrast microscopy (DIC; Figure 2b and top panels in Figure 2a’,B’; Fan et al., 2020; Redhai et al., 2016). DCGs are produced in secretory cells from a broad range of multicellular organisms, including insulin granules in pancreatic β‐cells and neuropeptide granules in neurons, though typically they are much smaller than in SCs (Gondré‐Lewis et al., 2012). They permit storage of proteins in an inert form until they are released during regulated secretion. Btl‐GFP also labels exosomes secreted from these compartments into the AG lumen (Figure 2a’’,b’’) (Fan et al., 2020).

In previous work, we found that in SCs, Btl‐GFP marks approximately 10 large non‐acidic compartments with diameter >2 μm (Fan et al., 2020). However, a stable fly line that we generated and used here, which permits inducible overexpression of Btl‐GFP, also marked multiple smaller (0.4–2.0 μm diameter) non‐acidic compartments (Figure S2a,c), so that the total number of labelled non‐acidic secretory compartments was 21 ± 8. Of these compartments, 67 ± 14% contained Btl‐GFP‐positive ILVs and 85 ± 9% contained DCGs (Figure S2a), confirming that most visible non‐acidic compartments are DCG‐containing compartments that also produce Rab11‐exosomes.

ILV formation inside large non‐acidic SC compartments requires the ESCRT‐0 Stam, the ESCRT‐I Vps28 and the ESCRT‐III Shrub (Fan et al., 2020). The roles of other ESCRT sub‐complex components in Rab11‐exosome formation and secretion were tested. Flies were generated that expressed RNAis targeting genes encoding members of all four ESCRT classes in adult SCs, using the GAL4/UAS system under temperature‐sensitive GAL80^ts^‐inducible control (Fan et al., 2020). Two genes in each ESCRT family were knocked down for 6 days following adult eclosion. As a control, an RNAi targeting the *rosy* gene was employed (Figure 2b, Figure S3a). *rosy* encodes the enzyme xanthine dehydrogenase required for normal eye colour, but has no known role in SC secretion.

SC‐specific knockdown of the *ESCRT‐0* genes (*Hrs* and *Stam*), the *ESCRT‐I* genes (*Vps28* and *TSG101*), the *ESCRT‐II* genes (*Vps25* and *Vps36*), and the *ESCRT‐III* genes (*shrub* [*Chmp4a‐c* in mammals] and *Chmp2)*, with two independent RNAis, in all cases significantly reduced the proportion of Btl‐GFP‐positive non‐acidic compartments, which contained fluorescent ILVs (Figure 2b‐e, 2g, 3A, 3B, 3H; Figure S3; Figure S4b). Furthermore, for almost all knockdowns, the numbers of Btl‐GFP puncta secreted into the lumen of the gland were significantly reduced compared to controls (Figures 2, 3; Figure S3h; Figure S4c).

Not all the compartment phenotypes produced by *ESCRT* knockdown were comparable, suggesting that the different classes of ESCRTs do not just function in a single linear ILV‐generating pathway. For example, the first step of exosome biogenesis involves the ESCRT‐0 proteins, Hrs and Stam (respectively Vps27 and Hse1 in yeast). As in mammalian cells (Razi & Futter, 2006), SCs depleted of ESCRT‐0 components, which cluster mono‐ubiquitinylated cargos at sub‐domains on the endosome membrane (Prag et al., 2007), had enlarged acidic compartments (Figure 2c, Figure S3b), when compared to controls (see also Corrigan et al., 2014; Fan et al., 2020).

For several *ESCRT* knockdowns, the number of Btl‐GFP‐positive, non‐acidic compartments increased significantly compared to controls, concomitant with an overall reduction in the size of these compartments (Figures 2b–f, 3b, 3g; Figure S3a–F; Figure S4a), suggesting that these ESCRTs have additional roles in compartment morphogenesis. The proportion of Btl‐GFP‐positive compartments that contained DCGs was also significantly reduced for many knockdowns (Figures S5a–E,J). Although this might be partly explained by the difficulty in detecting DCGs in smaller compartments, this does not fully account for our observations. For example, *Hrs* knockdown SCs lacked DCGs in almost all Btl‐GFP‐positive compartments, but this defect was not observed with knockdown of the other ESCRT‐0, *Stam*, which also contains many small compartments (Figure 2c’, Figure S3b’).

Compared to other core ESCRTs, knockdown of ESCRT‐II components *Vps25* and *Vps36* had a milder effect on Btl‐GFP‐labelled compartments (Figure 2e, Figure S3d). Compartment size and number were frequently more comparable to controls (Figure 2f, Figure S3f, Figure S4a), but for all knockdowns, there was still a reduction in the proportion of ILV‐forming compartments (Figure 2g, Figure S3g, Figure S4b). Notably, ILVs, when present, were often enlarged (Figure 2e’, Figure S3d’), suggesting that ESCRT‐II components might have a specific role in controlling the size of ILVs.

Overall, we conclude that similar to previous observations with late endosomal multivesicular compartments, ESCRT‐0, ‐I, ‐II and ‐III components are required to regulate the biogenesis of exosomes in large non‐acidic SC compartments, though the effects of *ESCRT‐II* knockdowns seem milder than for other ESCRTs. There is also a variable effect on DCG formation, even within the same ESCRT subclass, although knockdowns with the strongest effects on compartment size and ILV formation tend to have the most severe effect.

### 3.3 Accessory ESCRT‐III proteins are also involved in biogenesis of exosomes in large non‐acidic SC compartments

In light of our comparative human sEV proteomics data, we also investigated the roles of the accessory ESCRT‐III proteins in Rab11‐exosome biogenesis, as well as the AAA ATPase Vps4, which is recruited by the core and accessory ESCRT‐III proteins to promote scission of the ILV neck and recycling of the ESCRT complex (Azmi et al., 2008; Frankel et al., 2017; Henne et al., 2012; Rue et al., 2007; Teis et al., 2010).

Knockdown of *Vps4* had similar effects to knockdown of core ESCRT‐III proteins. Many small compartments, most without internal puncta, were observed (Figure 3f‐H, Supp Figures 4a, 4b). DCGs were absent, and exosome secretion was also reduced, though for one RNAi, this effect did not reach significance (Figure 3f’,f’’,i; Figure S4c, S5i,j).

Knockdown of the *accessory ESCRT‐III* genes also significantly affected ILV formation in Btl‐GFP‐positive SC compartments. In contrast, however, knockdown of *Chmp1*, *Chmp5* and *Ist1* did not significantly alter compartment number, but did reduce the proportion of compartments containing ILVs and decreased exosome secretion significantly (Figure 3c‐e, 3g‐i and Figure S4a‐C). DCG formation was also affected with *Chmp1* and *Ist1* knockdowns, though less severely than with many core *ESCRT* knockdowns (Figure S5f‐h, 5j).

In summary, we conclude that accessory ESCRT‐III proteins are required for normal exosome biogenesis in non‐acidic compartments of SCs. However, by comparison with *core ESCRT* knockdowns, reducing expression of these accessory proteins, particularly CHMP5 and IST1, has a much more subtle effect on the size and number of these large non‐acidic SC compartments.

### 3.4 ESCRT knockdowns do not affect non‐acidic compartment identity in *Drosophila* secondary cells

**FIGURE 4:**
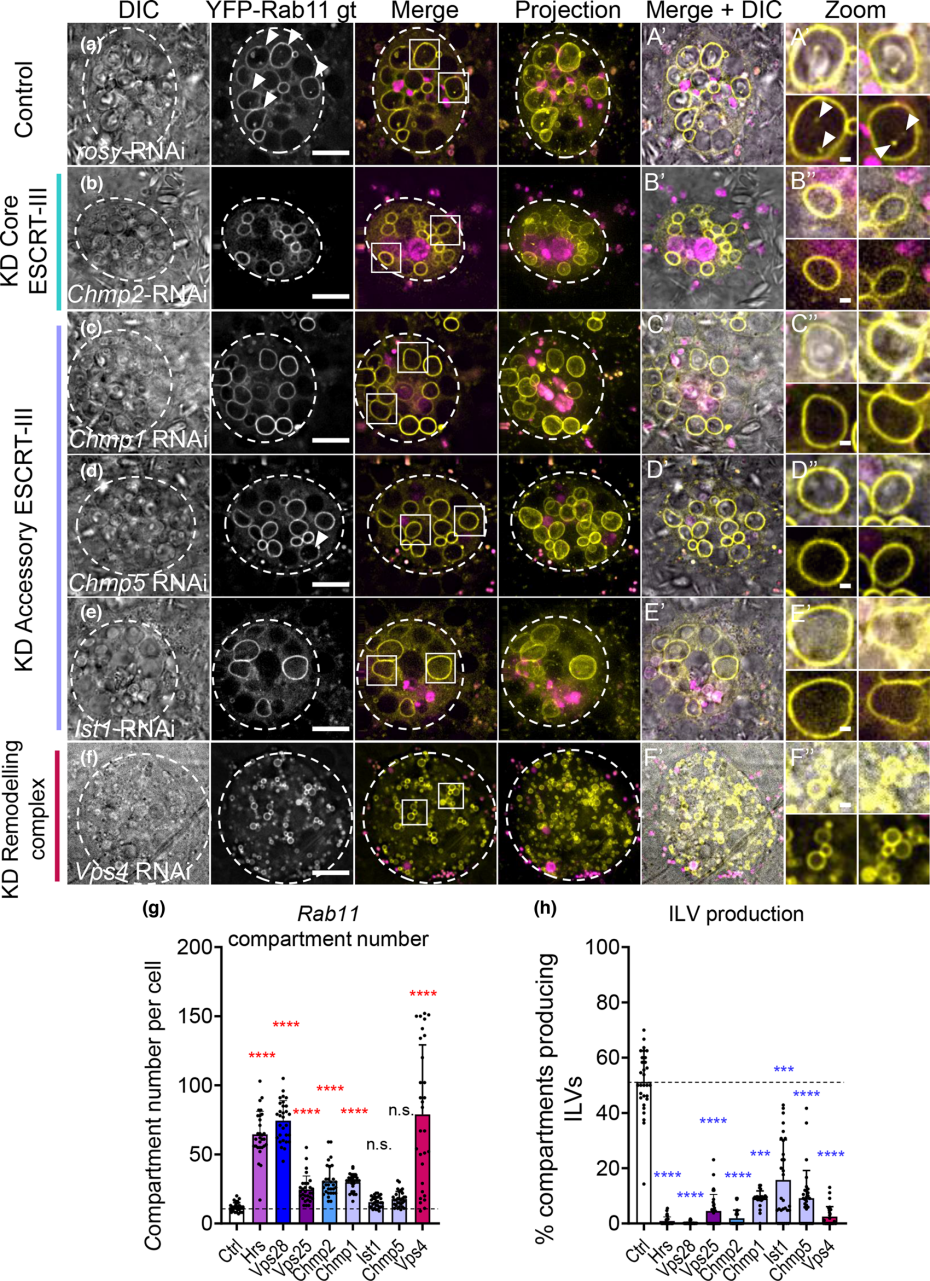
E*SCRT‐III* knockdown inhibits exosome biogenesis in non‐acidic SC compartments, but does not change compartment identity. Panels a‐f show basal wide‐field fluorescence views of living SCs expressing YFP‐Rab11 from its endogenous genomic location (yellow). SC outline approximated by dashed white circles. Acidic compartments are marked by LysoTracker^®^ Red (magenta). Boxed non‐acidic compartments are magnified in A’‐F’ Zoom. (a) Control SC expressing *rosy‐*RNAi. Rab11‐positive ILV puncta are present inside approximately 50% of Rab11‐compartments (arrowheads; Zoom). (b) In SCs expressing *Chmp2‐RNAi*, YFP‐Rab11‐compartment number is increased (g), but YFP‐Rab11‐positive ILVs are strongly reduced (h) and DCGs modestly reduced. (c) In SCs expressing *Chmp1*‐RNAi, YFP‐Rab11‐compartment number is increased (g). YFP‐Rab11‐positive ILVs are reduced (h), but DCGs are not affected. (d) In SCs expressing *Chmp5*‐RNAi, YFP‐Rab11‐compartment number is unchanged (g). YFP‐Rab11‐positive ILVs are reduced (h), but DCGs are not affected. (e) In SCs expressing *Ist1*‐RNAi, YFP‐Rab11‐compartment number is unchanged (g). YFP‐Rab11‐positive ILVs are reduced (h), but DCGs are not affected. (f) In SCs expressing *Vps4*‐RNAi, YFP‐Rab11‐compartment number is greatly increased (g). YFP‐Rab11‐positive ILVs and DCGs are greatly reduced (h). (g) Bar chart showing the number of non‐acidic YFP‐Rab11‐positive compartments with diameter > 0.4 μm per SC in control vs *ESCRT* knockdowns. *n* = 30. (h) Bar chart showing percentage of YFP‐Rab11 compartments containing YFP‐Rab11‐positive ILV puncta. *n* = 30.All data are from 6‐day‐old males shifted to 29°C at eclosion to induce transgene expression. Genotypes are: *w; P[w+, tub‐GAL80ts]/+; dsx‐GAL4; TI{TI}Rab11EYFP/+* with RNAi #1 lines for each gene. Scale bars in A‐F, 10 μm and in A’‐F’, 1 μm. Data were analysed by Kruskal‐Wallis test. ***p < 0.001 and ****p < 0.0001 relative to control. See also Figure S5 for images of gene knockdowns not depicted here, and Figure S4d, S4e for additional RNAi lines tested

One possible explanation for the reduction in ILV‐containing DCG compartments and associated defective DCG formation following *ESCRT* knockdown is that the identity of these compartments is altered by these genetic manipulations. To investigate this, a YFP‐tagged ‘gene trap’ marking Rab11 at the endogenous *Drosophila Rab11* locus (Dunst et al., 2015) was used to determine whether the defective compartments carry Rab11 at their limiting membrane and whether Rab11 marks a subset of ILVs, as is observed in wild‐type cells (Fan et al., 2020). In contrast to Btl‐GFP‐expressing cells, SCs in this gene trap line have essentially normal numbers of large compartments (12 ± 3 Rab11‐positive compartments of larger diameter than Btl‐GFP‐positive compartments and 3–4 LysoTracker‐positive LELs per cell; Figure 4a, Figure S2b,c). Their morphology was typically less disturbed by *ESCRT* knockdown than in SCs expressing other ILV markers (Figure 4, SF5, S6; Fan et al., 2020). 87 ± 11 % of Rab11‐positive compartments contain DCGs and 50 ± 12 % contain fluorescent ILVs, many of them around the DCG (Figure 4a and arrowheads in Figure 4a’; Figure S2b). Although these ILVs are secreted into the lumen of the gland, the low numbers preclude quantification of secretion with this marker.

SCs depleted of transcripts encoding the core ESCRT proteins and Vps4 often contained more Rab11‐positive compartments than controls (Figure 4b, 4f, 4g, Figures S4d, 6a‐i), but in all cases, a significantly reduced proportion of these compartments contained internal YFP‐Rab11 ILVs (Figure 4h, Figure S4e, S6j). Consistent with our observations using the Btl‐GFP marker, knockdowns producing very strong effects on ILV‐containing compartments typically reduced the proportion of DCG compartments significantly (Figure S5a’‐j’, S6k). However, other knockdowns that had a more limited effect on non‐acidic compartment number and size had little, if any, effect on DCG formation. In addition, the size of the largest acidic compartment was significantly increased in *ESCRT‐0* and *ESCRT‐II* knockdowns, but reduced in some other *ESCRT* knockdowns, such as *Vps28*, *shrb* and *Vps4*, which contain many small non‐acidic compartments (Figure S6l).

Therefore, reducing the levels of core ESCRT proteins does not appear to affect the identity of large non‐acidic compartments in SCs, but it does reduce their production of Rab11‐labelled ILVs and for all ESCRT groups except ESCRT‐II, it also affects compartment number and size, and DCG formation, mirroring the effects of these manipulations on Btl‐GFP‐expressing SCs.

Knockdown of the three *accessory ESCRT‐III* genes, *Chmp1*, *Chmp5* and *Ist1*, either did not affect the number of Rab11‐positive compartments or led to a modest, but significant, increase (Figure 4c‐e, 4g, SF4d). Acidic compartments and DCGs were mainly unaffected (Figure S5f’‐h’, 5j’, 6l). Importantly, knockdown of the accessory ESCRTs consistently reduced the proportion of Rab11‐compartments containing Rab11‐labelled puncta (Figure 4h), indicating that like the core ESCRTs, these genes are required for Rab11‐exosome biogenesis.

### 3.5 Accessory *ESCRT‐III* genes do not regulate deubiquitinylation of ILV cargos in SCs

**FIGURE 5:**
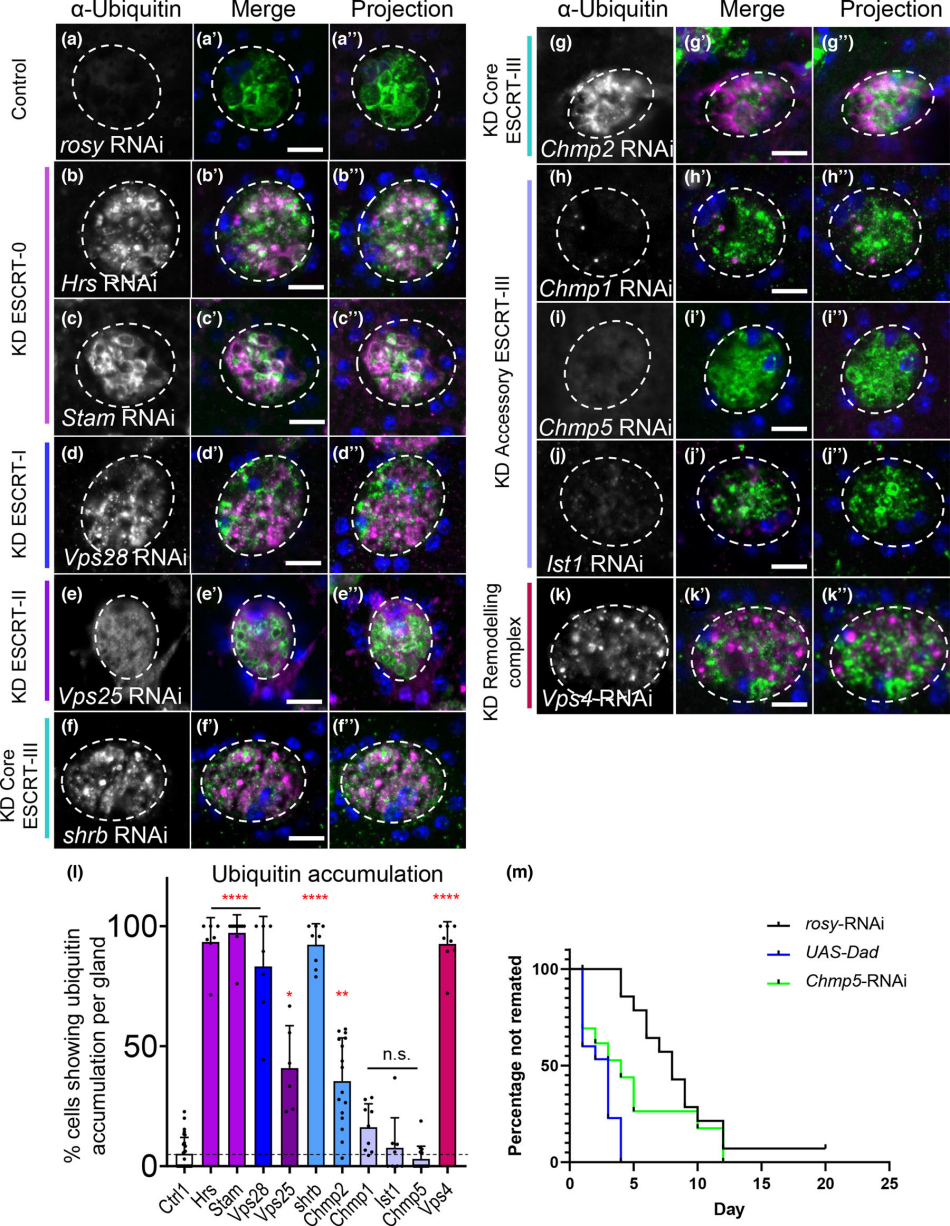
Accessory ESCRT‐III proteins are not required for processing of ubiquitinylated ILV cargos in SCs. Panels a‐k show confocal basal images of fixed SCs isolated from males expressing Btl‐GFP and selected *ESCRT*‐RNAis from eclosion onwards. SC outline approximated by dashed white circles. Ubiquitin (magenta), GFP (green) and DAPI (blue; cells are binucleate) staining is shown. Nuclear staining with anti‐ubiquitin is sometimes observed, even in controls, but appears non‐specific. (a) Control SC expressing *rosy*‐RNAi. Essentially no cytoplasmic accumulation of ubiquitin is observed in contrast to core ESCRT knockdowns (b‐g), where ubiquitin accumulates strongly in the cytosol and some Btl‐GFP‐positive compartments (colocalization in white; L). (b) SC expressing *Hrs*‐RNAi. (c) SC expressing *Stam*‐RNAi. (d) SC expressing *Vps28*‐RNAi. (e) SC expressing *Vps25*‐RNAi. (f) SC expressing *shrub*‐RNAi. (g) SC expressing *Chmp2*‐RNAi. (h) SC expressing *Chmp1*‐RNAi, as for other *accessory ESCRT‐III* knockdowns (i‐k), does not accumulate ubiquitin in the cytoplasm (l). (i) SC expressing *Chmp5*‐RNAi. (j) SC expressing *Ist1*‐RNAi. (k) SC expressing *Vps4*‐RNAi. (l) Bar chart showing proportion of SCs per gland that accumulate ubiquitin in the cytoplasm in control and *ESCRT* knockdowns. *n* ≥ 6 AGs. (m) Kaplan–Meier plot of remating for females initially mated with males expressing *rosy*‐RNAi, *Chmp5*‐RNAi or BMP antagonist Dad in adult SCs under esgF/O^ts^ control. Inhibiting BMP signalling, which suppresses secretion, or *Chmp5* expression is associated with more rapid remating of females. Similar effects were observed in three independent experiments. All data are from 6‐day‐old male flies shifted to 29°C at eclosion to induce transgene expression. Note that fixation required for ubiquitin visualisation disrupts SC subcellular morphology, when compared to live imaging. Genotypes are: *w; P[w^+^, tub‐GAL80^ts^]/+; dsx‐GAL4/P[w^+^, UAS‐btl‐GFP]* with *rosy‐* or RNAi #1 lines (a‐l) and *w; esg‐GAL4 tub‐GAL80^ts^ UAS‐FLP/CyO; UAS‐GFP_nls_ actin >* *FRT >* *CD2 >* *FRT >* *GAL4/TM6* with *rosy*‐RNAi, *chmp5‐*RNAi #2 or *UAS‐Dad* (m). Scale bars in A‐K, 10 μm. Ubiquitinylation data were analysed by Kruskal‐Wallis test. The remating data were analysed versus *rosy‐*RNAi control using a Gehan‐Breslow‐Wilcoxon test. *p < 0.05, **p < 0.01, ***p < 0.001 and ****p < 0.0001 relative to control

ESCRTs play a critical role in the degradation of ubiquitinylated proteins in the late endosome by clustering these molecules at the limiting membrane and then sequestering them into ILVs in a process involving deubiquitinylation (Mizuno et al., 2006). We hypothesised that the morphological defects observed in SCs expressing RNAis targeting the core ESCRTs might be partly explained by blockade of this process, which would lead to accumulation of ubiquitinylated ILV cargos. Indeed, in fixed and immunostained AGs from all core *ESCRT‐0*, *‐I* and *‐III*, and *Vps4* knockdown SCs expressing Btl‐GFP, ubiquitin accumulated at readily detectable levels throughout the cell (Figure 5b–g, k, l), unlike in control cells (Figure 5a). Consistent with the milder defects observed following knockdown of *ESCRT‐II* genes, ubiquitin accumulated to a more limited, but significant, extent in *Vps25* knockdown SCs. These results contrasted sharply with knockdowns of accessory *ESCRT‐III* genes (Figure 5a, 5l), where accumulation of ubiquitin was comparable to wild type cells (Figure 5h–j, 5l). These findings suggest that ESCRT‐dependent deubiquitinylation events on late endosomes are not strongly affected by accessory ESCRT‐III proteins, and that the latter proteins therefore selectively regulate Rab11‐exosome biogenesis. Consistent with our comparative human EV proteomics data (Tables S4, S5), our data also indicate that the generation of Rab11‐exosomes may not involve a ubiquitinylation/deubiquitinylation cycle for cargos, even though it requires ESCRT‐0 proteins that are thought to recruit these cargos (Prag et al., 2007).

We previously showed that the core ESCRT‐III protein Shrub is present in subdomains on the limiting membrane of Rab11‐compartments (Fan et al., 2020). To confirm that the ESCRT‐0 complex also concentrates around these compartments, as well as LELs, GFP‐tagged Hrs was expressed over a 24 h period in SCs. In addition to marking foci on the LEL limiting membrane, it localised to regions on the limiting membrane of at least one DCG‐containing compartment in most SCs (10/14 cells; Figure S6m), consistent with the ESCRT‐0 complex playing a direct role in Rab11‐exosome biogenesis.

We also tested how accessory ESCRT‐III knockdown affects the biological functions of SCs. Blocking BMP signalling, which is required for SC exosome secretion, or knocking down the ESCRT‐associated protein ALIX in SCs suppresses the inhibitory effect of seminal fluid on female receptivity to subsequent mating (Leiblich et al., 2012; Corrigan et al., 2014). Knockdown of *Chmp5* had a similar inhibitory effect on female receptivity to overexpressing the BMP signalling antagonist, Dad, in SCs (Figure 5 m), suggesting that the more targeted effect of *accessory ESCRT‐III* knockdown on Rab11‐exosome production is sufficient to block a specific reproductive signalling activity of SCs.

In summary, we conclude that although accessory ESCRT‐IIIs are required for Rab11‐exosome biogenesis and exosome‐mediated signalling, they do not appear to be essential for the processing of ubiquitinylated cargos during ILV formation in late endosomes.

### 3.6 The accessory *ESCRT‐III* gene *Chmp5* is selectively involved in Rab11a‐exosome biogenesis in human cells

**FIGURE 6:**
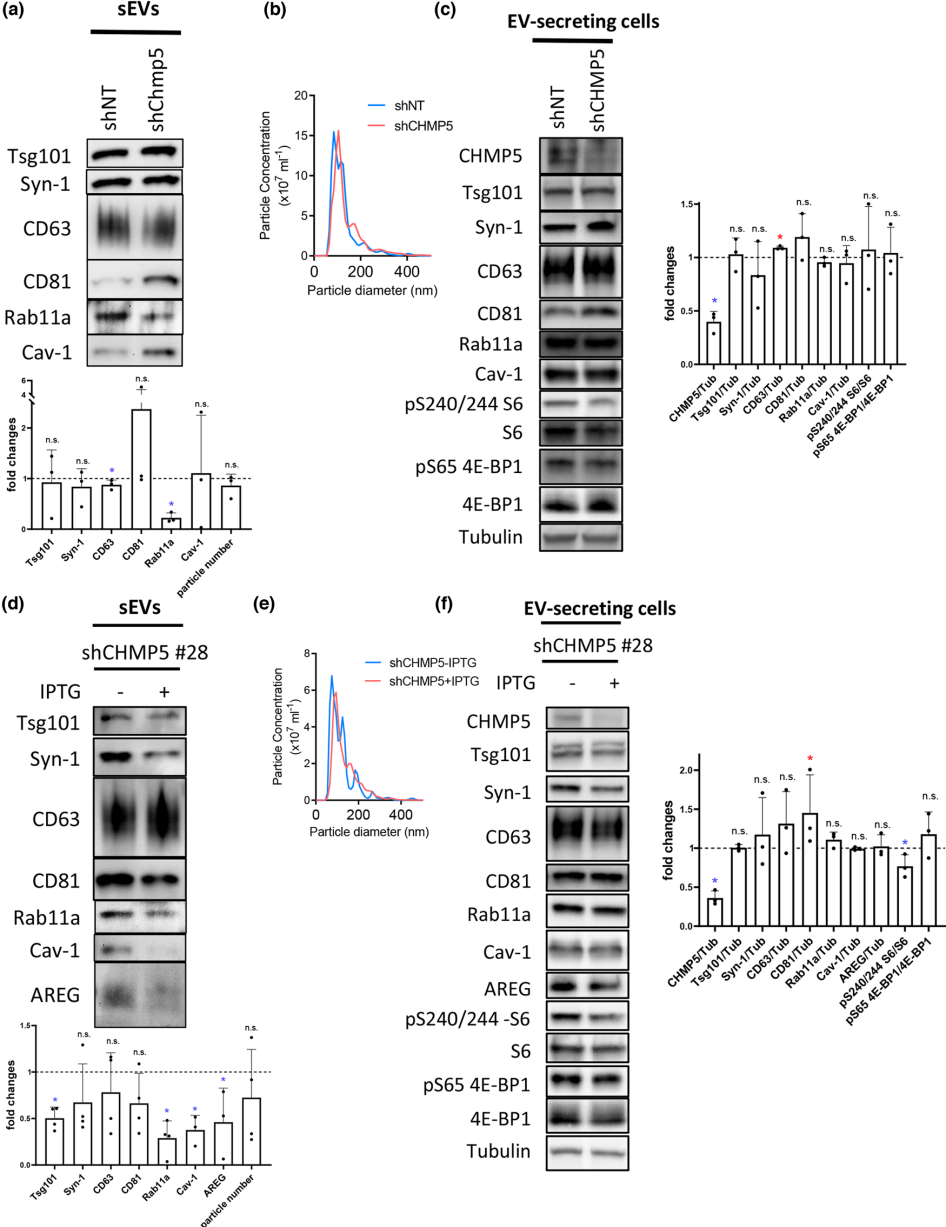
**Inducible knockdown of *CHMP5* selectively reduces Rab11a‐exosome production from glutamine‐depleted HCT116 cells**. (a) Western blot analysis of putative exosome proteins in sEV preparations isolated by size‐exclusion chromatography (SEC) from glutamine‐depleted (0.15 mM) EV‐secreting HCT116 cells transduced with a *CHMP5* (shCHMP5) compared to a non‐targeting (shNT) shRNA construct. Proteins were detected from gels with sample loading normalised to total cell lysate protein levels and the fold change in *CHMP5* knockdown cells determined. Note the more variable effects of knockdown on some of the sEV markers, but not Rab11a, in the bar chart. This is presumably the result of different levels of transient *CHMP5* knockdown. Data derived from three independent experiments. (B) Nanosight tracking analysis of sEVs for diluted samples (normalised to cell lysate protein levels) shows no change in particle size and number. (C) Western blot analysis of putative exosome proteins from glutamine‐depleted (0.15 mM) EV‐secreting HCT116 cells transduced with a *CHMP5* (shCHMP5) compared to a non‐targeting (shNT) shRNA construct shows selective reduction in CHMP5 protein levels. The activity of mTORC1 was assessed via phosphorylation of S6 and 4E‐BP1, using phospho‐specific antibodies and a pan 4E‐BP1 antibody. Bar chart shows protein levels normalised to tubulin. Data derived from three independent experiments. (D) Western analysis of sEV preparations isolated by SEC from HCT116 colorectal cancer cells, which contain a stable IPTG‐inducible *CHMP5* shRNA knockdown construct (clone #28; shCHMP5 #28), cultured in glutamine‐depleted (0.15 mM) conditions for 24 h. sEVs were collected from cells cultured in the absence (‐) or presence (+) of IPTG, both for 96 h previously and during the collection period. Putative exosome proteins were detected from gels with sample loading normalised to total cell lysate protein levels. Bar charts represent changes in levels of these putative exosome proteins relative to levels in the non‐IPTG‐induced sample. Data derived from five independent experiments. (E) Nanosight Tracking Analysis of sEVs for the shCHMP5 #28 samples produced in (D) show no change in particle size and number. (F) Western blot analysis of putative exosome proteins in EV‐secreting cells carrying a stable IPTG‐inducible *CHMP5* (shCHMP5 #28) shRNA construct, under glutamine‐depleted (0.15 mM) conditions, in the presence or absence of IPTG, reveals selective reduction in CHMP5 protein levels. Bar charts derived from three independent experiments. All data for bar charts were analysed by the Kruskal‐Wallis test: *p < 0.05; n.s. = not significant. Bars and error bars denote mean ± SD

To test whether the accessory *ESCRT‐III* genes also play a selective role in human Rab11a‐exosome formation, we investigated the effects of *Chmp5* knockdown on Rab11a‐exosome secretion from HCT116 cells. Cells were first transiently transfected with a constitutively expressed shChmp5 short hairpin RNA viral construct and in parallel, a non‐targeting shNT control. Under glutamine‐depleted conditions, the *Chmp5* knockdown construct significantly reduced CHMP5 protein levels in cell lysates (Figure 6c). The knockdown did not significantly affect the number of sEVs secreted, but it did have a strong and highly selective effect on the levels of Rab11a in these vesicles (Figure 6a,b).

**FIGURE 7:**
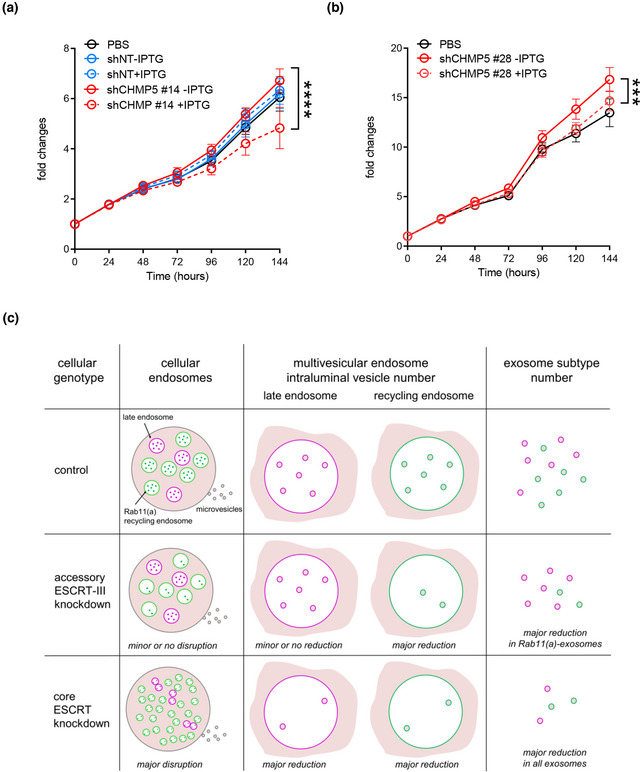
Inducible knockdown of *CHMP5* reduces the growth‐promoting effects of glutamine‐depletion‐induced sEVs on HCT116 recipient cells. (A) Growth curves in low (1%) serum are for HCT116 recipient cells cultured in glutamine‐depleted medium, pre‐treated with the sEV preparations from HCT116 cells carrying an inducible‐*CHMP5* (clone #14; red lines, solid without IPTG and dashed following IPTG induction of *CHMP5* knockdown) or an inducible‐non‐targeting (NT; blue lines, solid without IPTG and dashed following IPTG induction) shRNA knockdown construct compared to PBS (black solid line). Note that sEV preparations from this clone under glutamine depletion and in the absence of IPTG do not induce more growth than controls, suggesting that there is already some effect on the activity of these sEVs before induction of knockdown, probably because of leaky expression of the *CHMP5*‐shRNA. sEVs were separated by SEC. Growth curves were reproduced in three independent experiments and analysed by one‐way ANOVA. **** *p* ≤ 0.0001. (B) Growth curves in low (1%) serum are for HCT116 recipient cells cultured in glutamine‐depleted medium, pre‐treated with the sEV preparations, from HCT116 cells carrying an inducible‐*CHMP5* (clone #28; red lines, solid without IPTG and dashed following IPTG induction of *CHMP5* knockdown) shRNA knockdown construct compared to PBS (black solid line). sEVs were separated by SEC. Growth curves were reproduced in three independent experiments and analysed by one‐way ANOVA. *** ≤ 0.001. (C) Schematic model illustrating the mild effect of inducing *accessory‐ESCRT‐III* knockdown on the endosomal network compared to control cells, most clearly apparent in *Drosophila* secondary cells. This is accompanied by a major reduction in intraluminal vesicle formation within multivesicular endosomes labelled with the recycling endosomal marker Rab11(a) and selective reduction in exosomes secreted from these compartments, termed ‘Rab11(a)‐exosomes’, a subset of which are labelled with Rab11(a). In contrast, knockdown of the core ESCRT components leads to disruption of the endosomal network and also has a major impact on intraluminal vesicle formation from both late and recycling multivesicular endosomes

To produce cells that could be routinely used to knockdown CHMP5 protein levels in a controlled manner, we generated stable cell lines containing IPTG‐inducible versions of either shCHMP5 or shNT constructs. Two shCHMP5 lines were selected for further study, #14 and #28, because they produced a strong knockdown with IPTG induction, (Figure 6f; Figure S7a). Growth of these clones was not significantly affected by inducing knockdown either in glutamine‐replete or ‐depleted conditions (e.g., see Figure S8). In clones with this level of inducible knockdown, there was also reduced CHMP5 in non‐induced cells (Figure S7a; CHMP5 levels in shCHMP5 cells were 47.8 ± 14.9% of shNT cells in the absence of IPTG; *n* = 3, *p* < 0.05), presumably due to leaky expression of shCHMP5, which appeared to affect CHMP5‐dependent cellular functions even in the absence of IPTG induction (e.g., Figure 7a), and produced less pronounced selective effects on EV markers when compared to transient *CHMP5* knockdown (compare Figure 6a and 6D).

Mirroring the effects of non‐inducible *Chmp5* knockdown, both of the inducible clones had no significant effect on secreted sEV number following IPTG induction under glutamine‐depleted conditions (Figure 6e, Figure S7d). However, in both cases, levels of Rab11a in sEVs were consistently reduced after induction, while other exosome markers, which are thought to be present on late endosomal and Rab11a‐exosomes, were less reduced or not significantly changed (Figure 6d, Figure S7b). Therefore, as in *Drosophila* SCs, CHMP5 appears to be selectively involved in the regulation of exosomes containing Rab11a.

We previously found that sEV preparations produced by HCT116 cells under glutamine‐depleted conditions have increased growth‐promoting activity on naïve HCT116 cells when compared to sEVs produced under glutamine‐replete conditions (Fan et al., 2020). This requires the vesicle‐associated EGFR ligand Amphiregulin (AREG) and appears to involve trafficking through the Rab11a recycling endosomal pathway. Under glutamine‐depleted conditions, sEVs from both of the shCHMP5 inducible knockdown clones had either a modest (clone #28; Figure 7b), or no (clone #14; Figure 7a) growth‐promoting effect on HCT116 cells when collected under non‐induced, IPTG‐free conditions (Figure 7). This may be because of the leaky expression of the *CHMP5* knockdown construct (Figure S7a). However, in some experiments, we also found that sEVs from glutamine‐depleted shNT control cells had no significant growth‐promoting effect on HCT116 cells (Figure 7a), suggesting that in these specific experiments, a balance of growth‐promoting and growth‐inhibitory sEVs was being secreted. Nevertheless, in all cases, induction of *CHMP5* knockdown led to a significant reduction in HCT116 cell growth, which was not observed for shNT cells, suggesting that this genetic manipulation was removing growth‐promoting Rab11a‐exosomes, but not other vesicles, which presumably had a growth‐inhibitory effect (Figure 7a). Importantly, there appeared to be a significant reduction in sEV‐associated AREG protein following *CHMP5* knockdown, consistent with this interpretation (Figure 6d, Figure S7b), although the levels of AREG detected in control sEV preparations were low, presumably because of the leaky knockdown in these inducible cell lines under non‐induced conditions.

Taken together, these findings strongly suggest that CHMP5 selectively regulates the production of Rab11a‐exosomes and that these vesicles have a specific role in the AREG‐dependent, growth‐promoting activity of sEVs collected under glutamine‐depleted conditions.

## 4 DISCUSSION

We have recently identified Rab11a‐positive recycling endosomes as a source of exosomes, in addition to the well‐established late endosomal pathway. Exosomes from these two compartments carry different cargos, an observation strongly supported by our comparative sEV proteomics data. In cancer cells, Rab11a‐exosomes appear to have novel functions that may be involved in adaptive responses. In *Drosophila*, some of the core ESCRTs have previously been reported to control Rab11‐exosome biogenesis (Corrigan et al., 2014; Fan et al., 2020), mirroring their roles in forming late endosomal exosomes (Schuh & Audhya, 2014). Here we present data supporting a selective role for accessory ESCRT‐III proteins in Rab11(a)‐exosome production, but not in ubiquitin‐mediated late endosomal cargo degradation. We therefore propose that these proteins promote ILV formation via a mechanism that differs from late endosomes and does not involve ubiquitin.

### 4.1 Comparative sEV proteomics highlights different regulation and loading of Rab11a‐exosomes and late endosomal exosomes

Our previous studies revealed that switching HCT116 cells from glutamine‐replete to glutamine‐depleted conditions induces two‐ to three‐fold changes in specific exosome proteins, as the balance of exosome secretion switches towards Rab11a‐exosomes (Fan et al., 2020). By using TMT labelling in our comparative proteomics studies, we were able to detect less than two‐fold changes in protein levels, which were nonetheless statistically significant, in the five‐paired samples analysed, and therefore identified two groups of proteins that are at significantly higher levels either in Rab11a‐exosome or late endosomal exosome preparations. Analysis of these proteins revealed many GO terms that were enriched in one or other group (Tables S4, S5), most notably highlighting the increased levels of ESCRT‐III complex disassembly proteins, due to the enrichment of the accessory ESCRT‐III proteins. Western blot analysis confirmed that for some of these proteins there was a two‐ or more fold increase in sEVs isolated under glutamine‐depleted conditions, presumably because Rab11a‐exosomes are relatively more abundant when glutamine levels are reduced. Therefore, the comparative proteomics approach not only identifies cargos enriched in specific exosome subtypes, but as we have now shown, also highlights proteins that are involved in their differential regulation.

### 4.2 Core ESCRTs control Rab11‐recycling endosomal as well as late endosomal exosome biogenesis

All four ESCRT complexes have previously been implicated in the formation of multivesicular endosomes in yeast; *ESCRT* mutants produce endosomes without ILVs and so‐called ‘class E’ endosomal compartments with abnormal tubulation (Babst et al., 2002a; Raymond et al., 1992; Rieder et al., 1996). In higher organisms, there is evidence for ESCRT‐independent exosome production (Trajkovic et al., 2008); it has also been reported that cells depleted of components from any of the four core ESCRT complexes can generate CD63‐positive MVEs in metazoa via a ceramide‐dependent mechanism (Stuffers et al., 2009). However, exosome secretion from multivesicular late endosomes is heavily dependent on ESCRTs in multiple cell types (Colombo et al., 2014; Möbius et al., 2002; White et al., 2006), a process that leads to secretion of ILV‐associated receptors, ligands and cytosolic cargos with a wide range of signalling roles (Matusek et al., 2014). In this study, we have shown that ESCRT proteins from all ESCRT complexes are also needed for normal Rab11‐exosome biogenesis in fly SCs. Most of them also control the proper size and morphology of the large SC secretory compartments that make these exosomes, but have no effect on their Rab11 identity (see summary of data in Table S6). Although there is some variability in the extent of these morphological defects with different RNAis for the same gene and for knockdown of different *ESCRT*s in the same complex, the effects on exosome biogenesis are consistent and therefore unlikely to be the result of off‐target effects or novel gene function that is independent of the ESCRT complexes. Previous reports have demonstrated that ESCRT‐0 *Hrs* mutants induce the expansion of acidic endosomes (Razi & Futter, 2006). *Hrs* and *Stam* knockdown increase the size of the largest acidic compartment in SCs (Figure S6l), suggesting there are further parallels in ESCRT function between SCs and mammalian cells.

Core *ESCRT‐II* knockdowns produced the most variable phenotypes in our SC experiments. The proportion of ILV‐containing non‐acidic compartments was partially reduced by *ESCRT‐II* knockdown in SCs expressing either the exosome marker Btl‐GFP or YFP‐Rab11 (Figures 3, 4; Figures S3, S4, S6), but in Btl‐GFP‐expressing cells, the changes were often modest and frequently accompanied by the formation of large ILVs in some compartments. The effects on ubiquitin accumulation were also milder than with other core *ESCRT* knockdowns (Figure 5). It is unclear whether this can simply be explained by reduced knockdown of these genes in SCs or is the result of a mechanistic difference, although the fact that these weaker phenotypes are consistently seen with multiple knockdowns supports the latter hypothesis.

### 4.3 Dense‐core granule formation and Rab11‐exosome biogenesis appear to be functionally associated

Many of the more severe *ESCRT* knockdown phenotypes were not only characterised by a reduction in the proportion of ILV‐forming compartments, but also by effects on DCGs in non‐acidic compartments. This was most clearly observed in knockdowns of transcripts encoding the ESCRT‐0 component Hrs, the ESCRT‐I Vps28, the ESCRT‐III Shrub, and Vps4, which either appeared to block DCG formation entirely or in some compartments, led to formation of one or more small mis‐localised DCGs or ‘mini‐cores’ (Figure S5, S6k). With the Btl‐GFP marker, knockdowns for other core ESCRTs and the accessory ESCRT‐III proteins CHMP1 and IST1 also reduced DCG formation. Although absence of DCGs might be partly associated with the reduced size of non‐acidic compartments in these genetic backgrounds, leading to small cores that are not visible using DIC microscopy, DCGs were frequently absent or mislocalized even in larger compartments in these genetic backgrounds (e.g., Figure S5b, SB’, SC, SC’). Since DCGs are a common feature in cells that secrete proteins in a regulated fashion (Gondré‐Lewis et al., 2012), this association with ESCRT activity may have broad biological significance.

Currently we do not understand how ESCRT function and DCG biogenesis are linked, although it seems unlikely DCG formation is dependent on ILVs, because *Stam1* and *Tsg101* knockdowns strongly suppress ILV formation, but leave DCG number unaffected (Figure S6k). Interestingly, we recently reported a related mini‐core phenotype in SCs with reduced expression of the glycolytic enzyme, glyceraldehyde 3‐phosphate dehydrogenase 2 (GAPDH2; Dar et al., 2021). Multiple small dense cores, surrounded by ILVs, form in close proximity to the limiting membrane of non‐acidic SC compartments. Since extravesicular GAPDH2 appears to promote ILV clustering, one possible explanation for this phenotype is that mechanisms that cluster ILVs in Rab11‐compartments, so that they normally surround each core and link it to the limiting membrane, are also important for assembly of the large dense cores in SCs. Perhaps only some ESCRTs contribute to the events leading to this clustering process. Whether the same mechanisms are used in other cells with much smaller dense‐core granule compartments remains unclear.

### 4.4 Accessory *ESCRT‐III* knockdown selectively inhibits Rab11a‐exosome biogenesis

Accessory ESCRT‐III proteins have previously been postulated to be modulators of the vesicle scission activities of core ESCRT‐III components (Azmi et al., 2008; Rue et al., 2007). The CHMP1‐IST1 subcomplex is thought to facilitate the recruitment of Vps4 to ESCRT‐III (Agromayor et al., 2009; Rue et al., 2007) and the CHMP5‐Vta1 complex to enhance Vps4 activity (Shiflett et al., 2004; Azmi et al., 2006, 2008; Xiao et al., 2008). In the majority of previous studies, including in *Drosophila*, a single accessory *ESCRT‐III* knockdown or mutation appears to have either a subtle or no effect (Vaccari et al., 2009), and loss of an additional accessory ESCRT‐III protein is required to generate a strong phenotype (Bäumers et al., 2019). One explanation, which might be relevant to the link between Rab11a‐exosomes and nutrient deprivation, is that these accessory ESCRTs primarily interact with the ESCRT pathway under stress conditions, for example, when cells, like SCs, are highly metabolically active (Agromayor et al., 2009; Loncle et al., 2015; Shim et al., 2006), or at low temperatures (Bäumers et al., 2019).

In mammals, loss of *Chmp5* function is associated with early embryonic lethality (Shim et al., 2006). In *chmp5 ^‐/−^* primary embryonic cell cultures, enlarged late endosomes packed with vesicles are observed, suggesting that ILVs can still be made under these conditions. *Lip5/Vta1* and *chmp5* knockdowns in HeLa cells lead to EGFR accumulation (Ward et al., 2005), suggesting that aspects of endosomal trafficking are defective, but effects on ubiquitinylation and ILV formation were not investigated. In one example that parallels our findings, the accessory ESCRT‐III Ist1 is required to maintain cargo clustering and ILV formation in the *C. elegans* oocyte‐to‐embryo transition (Frankel et al., 2017). At this stage, MVE biogenesis appears rapid and the resulting ILVs are clustered, mirroring those seen in SCs. It will be interesting to investigate whether these MVEs are late endosomal or recycling endosomal in origin.

Our detailed analysis of ESCRT function in flies gave us the opportunity to determine whether accessory ESCRT‐III proteins have cellular roles in exosome biogenesis and how these roles differ from the well‐characterised core ESCRTs. Our data suggest that accessory ESCRT‐III proteins are selectively required for Rab11‐exosome biogenesis in *Drosophila* (Figures 3, 4) and this is mirrored by our analysis on human cells (Figure 6). In SCs, we observed two major differences in SC phenotypes associated with *accessory ESCRT‐III* knockdown when compared to knockdowns of most core ESCRTs. First, the size of Rab11 compartments and their morphology was only mildly affected by reduction of accessory ESCRT‐III proteins. Second, there was no obvious accumulation of ubiquitin. Ubiquitin accumulation following core *ESCRT* knockdown is thought to reflect the involvement of these ESCRTs in clustering ubiquitinylated cargos on the late endosomal limiting membrane and then deubiquitinylating them as ILVs form. Our *accessory ESCRT‐III* knockdown data suggest either that this ubiquitinylation/deubiquitinylation cycle can take place even in the absence of ILV formation, or more likely, that this cycle is not involved in Rab11‐exosome biogenesis and late endosomal ILVs continue to be generated in these knockdown cells.

In this regard, it is interesting to note that others have reported ESCRT‐dependent ILV biogenesis events that do not require ubiquitinylation. Most notably, stress‐internalised EGFR is trafficked to an alternative form of MVEs in human cells that is ESCRT‐dependent, but does not require ubiquitin for EGFR trafficking into ILVs (Tomas & Futter, 2015). In direct contrast, ubiquitin‐mediated endocytic degradation of the EGFR does not appear to require IST1 in human cells (Agromayor et al., 2009).

The potential involvement of core ESCRTs, like ESCRT‐0 Hrs, which is thought to interact with ILV cargos via its ubiquitin‐interacting motif, in ubiquitin‐independent ILV formation is intriguing. In other cells, ubiquitinylated Transferrin Receptors interact with Hrs and are sorted to the degradative pathway, whereas non‐ubiquitinylated endocytosed receptors fail to colocalize with Hrs and rapidly recycle to the cell surface (Matusek et al., 2019; Raiborg et al., 2001). Since Rab11‐exosomes are generated in recycling compartments, could their cargos be recruited via a different mechanism, for example, via a ubiquitin‐like protein (Hochstrasser, 2009)? In this regard, human CHMP4B (equivalent to *Drosophila* Shrub), CHMP1A and CHMP5 have been shown to directly interact with the SUMO‐conjugating enzyme Ubc9 (Tsang et al., 2006). Therefore, sumoylation is one loading mechanism that requires further investigation, particularly because it has been linked to recruitment of specific cargos into exosomes (Villarroya‐Beltri et al., 2013). Overall, we conclude that biogenesis of Rab11(a)‐exosomes requires an ESCRT‐dependent mechanism that unlike late endosomal exosomes, is ubiquitin‐independent, but also more reliant on accessory ESCRT‐III proteins, which consequently accumulate in this exosome subtype. At least in some cases, this novel biogenesis mechanism appears to be induced by specific cellular stresses and produces exosomes with specialised functions.

### 4.5 Tumour‐derived Rab11a‐exosomes have particularly potent signalling activity

Our *Chmp5* knockdown studies in both flies and human cancer cell lines suggest that Rab11a‐exosomes perform specialised physiological and pathological functions. In flies, the components of seminal fluid produced by the accessory gland, including the protein Sex Peptide (Kubli, 2003) and secretory products of SCs (Corrigan et al., 2014) have previously been shown to affect female behaviour, so she rejects males that subsequently attempt to mate with her. Knockdown of *Chmp5* in SCs inhibits this function, suggesting that Rab11a‐exosomes are important players in this mechanism, although the molecular cargos involved are yet to be identified.

Knockdown of *Chmp5* in glutamine‐depleted colorectal HCT116 cells leads to a selective reduction in the Rab11a marker in sEV preparations and a more modest reduction of several other exosome markers, consistent with our hypothesis that accessory ESCRT‐III proteins selectively regulate exosomes produced from Rab11a‐positive compartments. Importantly, there is little, if any, reduction in secreted sEV number, suggesting that Rab11a‐exosomes make up only a small proportion of secreted sEVs under these conditions. Nevertheless, their loss significantly inhibits the growth‐promoting activities of these vesicle preparations. Previously we have shown that AREG on these vesicles can stimulate cell growth at concentrations that are thousands of times lower than for soluble AREG (Fan et al., 2020). Therefore, although Rab11a‐exosomes may represent a relatively small population of secreted sEVs, they appear to be highly potent, perhaps because they have been assembled in the recycling endosomal pathway rather than the degradative late endosomal system. By contrast, Rab11‐exosomes from SCs appear to represent a much larger proportion of secreted vesicles. They also appear essential for exosome‐mediated signalling, which in this case controls female reproductive behaviour. One interesting implication of these findings is that it may be critical to select the appropriate subtype of exosome for bio‐delivery applications to optimise activity, therefore highlighting the importance of identifying subtype‐specific cargos for optimal loading.

We have previously proposed that stress‐induced Rab11a‐exosomes are likely to be involved in tumour adaptation mechanisms (Fan et al., 2020). Some accessory ESCRT‐IIIs have already been associated with human cancer: Ist1 overexpression (OLC1 in humans, also known as KIAA0174) has been observed in lung, breast and colorectal cancer as well as oesophageal squamous cell carcinoma (Li et al., 2014; Liu et al., 2014; Ou‐Yang et al., 2014; Yuan et al., 2008), and CHMP1A levels are reported to be increased in renal cell and pancreatic carcinomas (Li et al., 2008; Mochida et al., 2012). It is possible that selective inhibition of Rab11a‐exosome biogenesis by interfering with accessory ESCRT‐III function in such tumours could block adaptive responses without preventing other important events, such as the ubiquitin‐mediated degradation of growth factor receptors in cancer cells.

We conclude that accessory ESCRT‐III proteins selectively regulate Rab11‐ and Rab11a‐exosome formation via an ESCRT‐dependent mechanism, which does not appear to be dependent on a ubiquitinylation/deubiquitinylation cycle for cargo loading. Targeting this selective mechanism in other cell types should help to unravel the specific physiological functions of these vesicles and blocking these highly potent stress‐induced exosomes could provide a new route to halt cancer progression.

## AUTHOR CONTRIBUTIONS

**Pauline P. Marie**: Conceptualization; Data curation; Formal analysis; Investigation; Methodology; Project administration; Resources; Supervision; Validation; Visualization; Writing—original draft; Writing—review & editing. **Shih‐Jung Fan**: Conceptualization; Data curation; Formal analysis; Investigation; Methodology; Resources; Validation; Writing—original draft; Writing—review & editing. **John Mason**: Conceptualization; Data curation; Formal analysis; Investigation; Methodology; Writing—review & editing. **Adam Wells**: Conceptualization; Data curation; Formal analysis; Investigation; Methodology; Validation; Writing—review & editing. **Cláudia C. Mendes**: Methodology; Resources; Writing—review & editing. **S. Mark Wainwright**: Methodology; Resources; Writing—review & editing. **Sheherezade Scott**: Data curation; Formal analysis; Methodology; Writing—review & editing. **Roman Fischer**: Conceptualization; Data curation; Formal analysis; Funding acquisition; Investigation; Methodology; Project administration; Supervision; Writing—review & editing. **Adrian L. Harris**: Funding acquisition; Project administration; Supervision; Writing—review & editing. **Clive Wilson**: Conceptualization; Funding acquisition; Project administration; Supervision; Writing—original draft; Writing—review & editing. **Deborah C. I. Goberdhan**: Conceptualization; Funding acquisition; Project administration; Supervision; Writing—original draft; Writing—review & editing.

## CONFLICT OF INTEREST STATEMENT

The authors declare that they have no conflicts of interest in relation to this work.

## Supporting information

Supporting Information

Supporting Information

Supporting Information

Supporting Information

Supporting Information

Supporting Information

Supporting Information

Supporting Information

Supporting Information

Supporting Information

Supporting Information

Supporting Information

Supporting Information

Supporting Information

Supporting Information
