# Correction to “Accessory ESCRT‐III proteins are conserved and selective regulators of Rab11a‐exosome formation”

**DOI:** 10.1002/jev2.12321

**Published:** 2023-05-02

**Authors:** Shih‐Jung Fan

**Affiliations:** ^1^ Department of Physiology Anatomy and Genetics University of Oxford Oxford UK; ^2^ Department of Life Sciences National Central University Taoyuan City Taiwan

1

Marie, P. P., Fan, S.‐J., Mason, J., Wells, A., Mendes, C. C., Wainwright, S. M., Scott, S., Fischer, R., Harris, A. L., Wilson, C., & Goberdhan, D. C. I. (2023). Accessory ESCRT‐III proteins are conserved and selective regulators of Rab11a‐exosome formation. *Journal of Extracellular Vesicles*, 12, e12311. https://doi.org/10.1002/jev2.12311

In the originally published article, both of author Shih‐Jung Fan's affiliations were not included. Both affiliations are shown below. This has been updated in the online version of the article.

We apologize for this error.

Shih‐Jung Fan^1,2^

^1^Department of Physiology Anatomy and Genetics, University of Oxford, Oxford, UK

^2^Department of Life Sciences, National Central University, Taoyuan City, Taiwan

