# Supplementary material for: Accessory ESCRT‐III proteins are conserved and selective regulators of Rab11a‐exosome formation
Source: J Extracell Vesicles. 2023 Mar 5;12(3):12311. doi: 10.1002/jev2.12311 (PMC9986085; doi:10.1002/jev2.12311)

# Supplementary Figures

### **Supp. Figure 1. Accessory ESCRT-III components are not increased in HCT116 cells in glutamine-depleted compared to glutamine-replete conditions or concentrated in secreted sEVs (related to Fig. 1).**

### (A) Western blot analysis of putative exosome markers in glutamine-depleted (0.15 mM) sEV-secreting cells compared to cells cultured in glutamine-replete (2.0 mM) conditions shows no significant change in the levels of accessory ESCRT-III components, CHMP1A, CHMP1B and IST1. The activity of mTORC1 was assessed via phosphorylation of S6 and 4E-BP1, using phospho-specific antibodies and a pan 4E-BP1 antibody. Bar chart shows protein levels normalised to tubulin. Data derived from three independent experiments and analysed by the Kruskal-Wallis test. Significantly changed levels are denoted by a blue (decreased) and red (increased) asterisk. *P < 0.05; n.s. = not significant.

(B) Western blot of putative exosome and non-exosome proteins in cell lysates and sEV preparations from HCT116 cells cultured under glutamine-depleted conditions reveals that accessory ESCRT-III proteins CHMP1A, CHMP1B and IST1 are present, but not enriched in the secreted sEVs relative to cell lysate protein levels. sEVs were separated by differential ultracentrifugation.

(C) Western blot of putative exosome proteins in sEV preparations from HCT116 cells cultured under glutamine-depleted conditions following incubation in the presence or absence of Proteinase K and/or Triton^®^ X-100. Note that only CD81 is digested by Proteinase K in the absence of Triton X-100, intraluminal Tsg101 and Syn-1 are only fully digested in the presence of detergent, while as previously shown (Fan et al., 2020), CD63 is resistant to digestion, even after detergent treatment, presumably because of its strong membrane association. CHMP1B is also resistant to protease digestion, even in the presence of detergent, suggesting that it is also strongly associated with membranes. Some proteins, like CHMP1B, migrate more slowly in Proteinase K-treated samples, perhaps because of the addition of PMSF in ethanol to these samples, which was required to terminate protease digestion. This experiment was repeated twice with similar results. sEVs were separated by differential ultracentrifugation. The dark signals at the bottom corners of the CHMP1B western blot are produced by over-exposed markers in adjacent lanes.

### **Supp. Figure 2. Large non-acidic compartments of *Drosophila* SCs form DCGs and exosomes when expressing either Btl-GFP or a *YFP-Rab11* gene trap.**

(A) Bar chart showing average number of Btl-GFP-positive compartments per cell, the proportion containing DCGs and Btl-GFP-positive ILVs, and average size of compartments per cell. n = 30 SCs.

(B) Bar chart showing average number of YFP-Rab11 compartments per cell, the proportion containing DCGs and YFP-Rab11-positive ILVs, and average size of compartments per cell. n = 30 SCs.

(C) Individual non-acidic compartment sizes in SCs expressing the two markers. n = 30 SCs.

All data are from 6-day-old males shifted to 29°C at eclosion to induce expression of transgenes. Genotypes are: *w; P[w^+^, tub-GAL80^ts^]/+; dsx-GAL4/P[w^+^, UAS-btl-GFP]* or *w; P[w^+^, tub-GAL80^ts^]/+; dsx-GAL4w; TI{TI}Rab11EYFP/+* with *UAS-rosy-RNAi* expression.

### **Supp. Figure 3. ESCRTs regulate exosome biogenesis in non-acidic compartments of *Drosophila* SCs (related to Figs. 2 and 3).**

Panels A-E show basal wide-field fluorescence views of living SCs from 6-day-old males expressing the GFP-tagged form of Breathless (Btl-GFP; green) and a selected RNAi from eclosion onwards. SC outline approximated by dashed white circles. Acidic compartments are marked by LysoTracker^®^ Red (magenta). Boxed non-acidic compartments are magnified in A’-E’.

(A) Control SC expressing *rosy*-RNAi construct. Btl-GFP-positive ILVs are visible in many non-acidic compartments (arrowheads; A’) and as puncta in the projection of the AG lumen (A’’).

(B) SC expressing *Stam*-RNAi. Acidic compartment size is increased. Btl-GFP-positive ILVs and puncta in the AG lumen are reduced, but DCGs are still present (B’).

(C) SC expressing *TSG101*-RNAi has less Btl-GFP-positive ILVs and secreted puncta appear reduced, though the latter effect is not significant.

(D) SC expressing *Vps36*-RNAi has a lower proportion of non-acidic compartments containing Btl-GFP-positive puncta. ILVs are often enlarged and exosome secretion is reduced.

(E) SC expressing *shrb-*RNAi contains many small non-acidic compartments, very few of which contain Btl-GFP-positive ILVs and DCGs, and exosome secretion is reduced.

(F) Bar chart showing the number of non-acidic Btl-GFP-positive compartments per SC with diameter > 0.4 µm in control versus *ESCRT-0*, *-I*, and *-II* knockdowns (n = 30 SCs).

(G) Bar chart showing percentage of Btl-GFP compartments containing Btl-GFP-positive ILVs (n = 30 SCs).

(H) Bar chart showing number of Btl-GFP fluorescent puncta in the lumen of AGs with *ESCRT* knockdown compared to control. n ≥ 10.

Genotypes are: *w; P[w^+^, tub-GAL80^ts^]/+; dsx-GAL4,P[w^+^, UAS-btl-GFP]/+* with *UAS-rosy-RNAi* (A), *UAS-Stam-RNAi*-#1 (B), *UAS-TSG101-RNAi-#*1 (C), *UAS-Vps36-RNAi*-#1 (D),*UAS-shrb-RNAi*-#1 (E). Scale bars in A-E and AG lumen, 10 µm and in A’-E’, 1 µm.

### **Supp. Figure 4. ESCRTs regulate exosome biogenesis in non-acidic compartments of SCs (related to Figs. 2, 3 and 4).**

Quantification of live SCs expressing a second RNAi line (#2) for all *ESCRT* genes shown in Figs 2, 3, 4, EV2 and EV4.

(A) Bar chart showing the number of non-acidic Btl-GFP-positive compartments with diameter > 0.4 µm per SC in control vs *ESCRT-0*, *-I*, *-II*, *-III* and *accessory ESCRT III* knockdowns. n = 30.

(B) Bar chart showing percentage of Btl-GFP-positive compartments containing fluorescent ILVs for control and *ESCRT* knockdowns. n = 30.

(C) Bar chart showing number of Btl-GFP fluorescent puncta in AG lumen following *ESCRT* knockdown compared to control SCs. Note that *Vps28*, *Vps25* and *Vps36* RNAis do not significantly reduce secreted puncta, but for *Vps28* and *Vps36* RNAis, there is a strong reduction in the mean number of puncta, but this failed to reach significance because of the comparisons between multiple knockdowns involved in each analysis. n ≥ 10.

(D) Bar chart showing the number of non-acidic YFP-Rab11-positive compartments with diameter > 0.4 µm per SC in control vs *ESCRT* knockdowns. n = 30.

(E) Bar chart showing percentage of YFP-Rab11 compartments containing YFP-Rab11-positive ILVs for control and *ESCRT* knockdowns.

All data are from 6-day-old males shifted to 29°C at eclosion to induce expression of transgenes. Genotypes are: *w; P[w^+^, tub-GAL80^ts^]/+; dsx-GAL4, P[w^+^, UAS-btl-GFP]/+* with *UAS-rosy-RNAi*, and RNAi #2 lines for each *ESCRT*. Data were analysed by Kruskal-Wallis test. Significantly changed levels are denoted by a blue (decreased) and red (increased) asterisk. *p < 0.05, **p < 0.01, ***p < 0.001, and ****p < 0.0001 relative to control.

### **Supp. Figure 5. Knockdown of principal ESCRT components disturbs DCG formation (related to Figs. 2, 3 and 4).**

Transverse images of non-acidic compartments are shown for *rosy*-RNAi control versus *ESCRT-0*, *-I*, *-II*, *–III* and *accessory ESCRT-III* knockdowns. DIC images of three compartments of decreasing size are shown per genotype (grey-scale) with Btl-GFP (A-I) and YFP-Rab11 (A’-I’) employed as markers for each knockdown. Yellow arrowheads indicate intact dense-core granules and red arrowheads show fragmented/displaced DCGs inside non-acidic compartments.

(A, A’) SC expressing *rosy-*RNAi.

(B, B’) SC expressing *Hrs*-RNAi.

(C, C’) SC expressing *Vps28-*RNAi.

(D, D’) SC expressing *Vps25*-RNAi.

(E, E’) SC expressing *Chmp2*-RNAi.

(F, F’) SC expressing *Chmp1*-RNAi.

(G, G’) SC expressing *Chmp5*-RNAi.

(H, H’) SC expressing *Ist1*-RNAi.

(I, I’) SC expressing *Vps4*-RNAi.

(J, J’) Bar charts showing proportion of YFP-Rab11 compartments with normal centrally located DCGs in each SC with Btl-GFP (J) and YFP-Rab11 (J’) employed as markers. Normal DCGs are visible in cells following *rosy-*RNAi expression (A,A’) and for *Chmp1*-RNAi (F’), *Chmp5*-RNAi (G,G’) and *Ist1*-RNAi (H,H’). *Hrs*-RNAi (B,B’), *Vps28*-RNAi (C,C’) and *Vps4-*RNAi (I,I’) most strongly suppress DCG formation: fragmented or displaced dense cores can sometimes be seen at the limiting membrane of the compartment.

Genotypes are: w; *P[w^+^, tub-GAL80^ts^]/+; dsx-GAL4, P[w^+^, UAS-btl-GFP]/+* with *UAS-rosy-RNAi* knockdown construct (A), *UAS-Hrs-RNAi*-#1 (B), *UAS-Vps28-RNAi*-#1 (C), *UAS-Vps25-RNAi*-#1 (D), *UAS-Chmp2-RNAi*-#1 (E), *UAS-Chmp1-RNAi*-#1 (F), *UAS-Chmp5-RNAi*-#1 (G), *UAS-Ist1-RNAi*-#1 (H) and *UAS-Vps4-RNAi*-#1 (I) and *w; P[w^+^, tub-GAL80^ts^]/+; dsx-GAL4; TI{TI}Rab11EYFP/+* with the same transgenes (A’-I’). Scale bars in A-I’ 5 µm.

### **Supp. Figure 6. *ESCRT* knockdown inhibits exosome biogenesis in non-acidic SC compartments, but does not change compartment identity (related to Fig. 4).**

Panels A-H show basal wide-field fluorescence views of living SCs expressing YFP-Rab11 from its endogenous genomic location (yellow). SC outline approximated by dashed white circles. Acidic compartments are marked by LysoTracker^®^ Red (magenta, Merge). Boxed non-acidic compartments are magnified in A’-H’.

(A) Control SC expressing *rosy-*RNAi. YFP-Rab11-positive ILV puncta are observed inside nearly 50% of Rab11-compartments (arrowheads; A’).

(B) SC expressing *Hrs*-RNAi

(C) SC expressing *Stam-*RNAi

(D) SC expressing *Vps28*-RNAi

(E) SC expressing *TSG101-*RNAi

(F) SC expressing *Vps25*-RNAi

(G) SC expressing *Vps36-*RNAi

(H) SC expressing *shrub-*RNAi

(I) Bar chart showing the number of non-acidic YFP-Rab11-positive compartments with diameter > 0.4 µm per SC in control vs *ESCRT* knockdowns. n = 30.

(J) Bar chart showing percentage of YFP-Rab11 compartments containing YFP-Rab11-positive ILV puncta. n = 30.

(K) Bar chart showing proportion of YFP-Rab11 compartments with normal centrally located DCGs in each SC. Note that *Stam1* knockdown has a strong effect on ILV formation, but not DCG biogenesis. n = 30.

(L) Bar chart showing number of acidic compartments per SC and maximum area of the largest acidic compartment in µm². n = 30.

(M) Basal view through a living SC from a 6-day-old male, following a 24 h pulse of *UAS-Hrs-GFP* transgene expression at 29ºC. In addition to punctate localisation at the surface of LELs (marked by dotted white circles in top right image and arrowheads in bottom right-hand image in Zoom), which are marked by the vital dye LysoTracker^®^ Red (magenta), Hrs-GFP (green) is found in foci (arrowheads in bottom left-hand image in Zoom) at the membrane of at least one non-acidic compartment (arrowhead in middle images). Images were acquired by wide-field microscopy. Genotype for this experiment is *w; P[w^+^, tub-GAL80^ts^]/+; dsx-GAL4 P[w^+^, UAS-Hrs-GFP]/+.*

All data, except for M, are from 6-day-old males shifted to 29°C at eclosion to induce transgene expression. Genotypes are: *w; P[w+, tub-GAL80^ts^]/+; dsx-GAL4; TI{TI}Rab11EYFP/+* with RNAi #1 lines for each gene. Scale bars in A-H, 10 µm and in A’-H’, 1 µm. Data were analysed by Kruskal-Wallis test. *p < 0.05, **p < 0.01, ***p < 0.001, ****p < 0.0001 relative to control.

**Supp. Figure 7.** ***Chmp5* knockdown in an IPTG-inducible clone reduces the production of Rab11a-exosomes under glutamine-depleted conditions (related to Fig. 6).**

(A) Western blot analysis of putative exosome proteins in sEV-secreting cells under glutamine-depleted (0.15 mM) conditions and carrying stable IPTG-inducible non-targeting (shNT) or *CHMP5* (shCHMP5 #14) shRNA constructs in the presence or absence of IPTG. Selective reduction in CHMP5 protein levels is shown by shCHMP5#14, but note that CHMP5 levels are reduced in shCHMP5 #14 cells, even in the absence of IPTG. The activity of mTORC1 was assessed via phosphorylation of S6 and 4E-BP1, using phospho-specific antibodies and a pan 4E-BP1 antibody. Bar chart shows protein levels normalised to tubulin.

(B) Western analysis of small extracellular vesicle (sEV) preparations isolated by size-exclusion chromatography (SEC) from HCT116 colorectal cancer cells cultured in glutamine-depleted (0.15 mM) conditions for 24 h, following transduction with an IPTG-inducible non-targeting sh(NT) construct or using a stable clone containing a *CHMP5* shRNA knockdown construct (clone #14; shCHMP5 #14). sEVs were collected from cells cultured both for 96 hours previously and during the collection period in the absence (-) or presence (+) of IPTG. Putative exosome proteins were detected from gels with sample loading normalised to total cell lysate protein levels. Bar charts represent changes in levels of putative exosome proteins normalised to cell lysate and then to their levels in the shNT non-induced control. Data derived from three independent experiments and analysed by the Kruskal-Wallis test: *P < 0.05; n.s. = not significant. Bars and error bars denote mean ± SD.

(C) Nanosight Tracking Analysis of sEV size and number for the shNT samples produced in (B).

(D) Nanosight Tracking Analysis of sEV size and number for the shCHMP5 samples produced in (B).

**Supp. Figure 8. *Chmp5* knockdown in an IPTG-inducible clone does not affect cell growth under glutamine-replete or -depleted conditions (related to Fig. 6).**

(A, B) Growth curves for the HCT116 clone #14 carrying an inducible-*CHMP5* shRNA knockdown construct in the presence of the inducer IPTG (yellow lines) or in its absence (grey lines) under glutamine-replete (A) and -depleted (B) conditions. The cells were treated in the same way as for sEV collection, namely IPTG was first added at Day 0, the cells were replated ± IPTG at 500 cells/well on Day 3 and growth medium was replace by EV collection buffer ± IPTG on Day 5. Growth curves were analysed by paired t-test and reproduced in three independent experiments and. There was no significant effect of IPTG addition on growth.

Supplementary Tables

### **Supp. Table 1. Proteins increased in sEV preparations from glutamine-depleted HCT116 cells versus glutamine-replete cells.**

Table lists the 48 proteins that are increased in proteomics analysis of sEV preparations from glutamine-depleted HCT116 cells, ranked according to mean fold change. Accessory ESCRT-III proteins (IST1, CHMP1BA, CHMP1B and CHMP5) are shaded in green.

### **Supp. Table 2. Proteins decreased in sEV preparations from glutamine-depleted HCT116 cells versus glutamine-replete cells.**

Table lists the 87 proteins that are decreased in proteomics analysis of sEV preparations from glutamine-depleted HCT116 cells, ranked according to mean fold change.

### **Supp. Table 3. GO terms associated with multivesicular endosomes, exosomes and virus secretion are highly enriched in the HCT116 sEV proteomic analysis.**

Table lists most enriched GO terms for the 683 proteins identified in all ten HCT116 sEV samples analysed by TMT-labelled proteomics, ranked by fold enrichment.

### **Supp. Table 4. Molecules associated with ESCRT complex disassembly are the most enriched among the proteins elevated in Rab11a-exosome-enriched sEV preparations.**

Table lists most enriched GO terms for the 48 proteins that are increased in proteomics analysis of sEV preparations from glutamine-depleted HCT116 cells versus glutamine-replete cells, ranked by fold enrichment. Note terms associated with ESCRT complex disassembly are most enriched (green shading); the identified molecules from this category are the accessory ESCRT-III proteins. These proteins have also been reported to be involved in cytokinesis in some cells, hence the enrichment for terms linked to mitosis.

### **Supp. Table 5. Molecules associated with ubiquitin-dependent endocytosis are highly enriched among the proteins reduced in the Rab11a-exosome-enriched sEV preparations.**

Table lists most enriched GO terms for the 87 proteins that are decreased in proteomics analysis of sEV preparations from glutamine-depleted HCT116 cells versus glutamine-replete cells, ranked by fold reduction. Note terms associated with ubiquitin-dependent endocytosis are among these (green shading), suggesting that this process is more associated with late endosomal exosome secretion.

### **Supp. Table 6. Summary of *ESCRT* knockdown phenotypes in SCs**

Table summarises the effects of specific knockdown (Kd) in fly SCs of the expression of different components of the ESCRT machinery, namely the core ESCRT-0 (Hrs, Stam), ESCRT-I (Vps28, Tsg101), ESCRT-II (Vps25, Vps36), ESCRT-III (Chmp2, Shrb) and accessory (Acc) ESCRT-IIII (Chmp1, Chmp5, Ist1) subcomplexes, plus the ATPase (Vps4). The following features were analysed: within the SC cytoplasm the accumulation of ubiquitinated (UBN) cargos, which not present in controls; and within the SC non-acidic endosomes, firstly, the presence of fluorescent intraluminal vesicles (ILVs), which are present in controls in about 65% (Btl-GFP overexpression) and 50% (YFP-Rab11 endogenous expression) of these compartments; and secondly, the presence of protein-rich dense core granules (DCGs), which are typically found in >80% of control compartments. For ILVs, ‘*Few*’ means most SC non-acidic compartments lack fluorescent ILVs. For DCGs, ‘*Few*’ means <10% of non-acidic compartments contain normal DCGs; ‘*Some*’ means between 10 and 50% of non-acidic compartments contain normal DCGs. * indicates that the absence of DCGs was dependent on the marker expressed in SCs (ie absent with Btl-GFP, but present with YFP-Rab11). ** indicates that the UBN cargos appeared to accumulate to a lesser extent than other cases shown. No data (nd) collection is indicated.


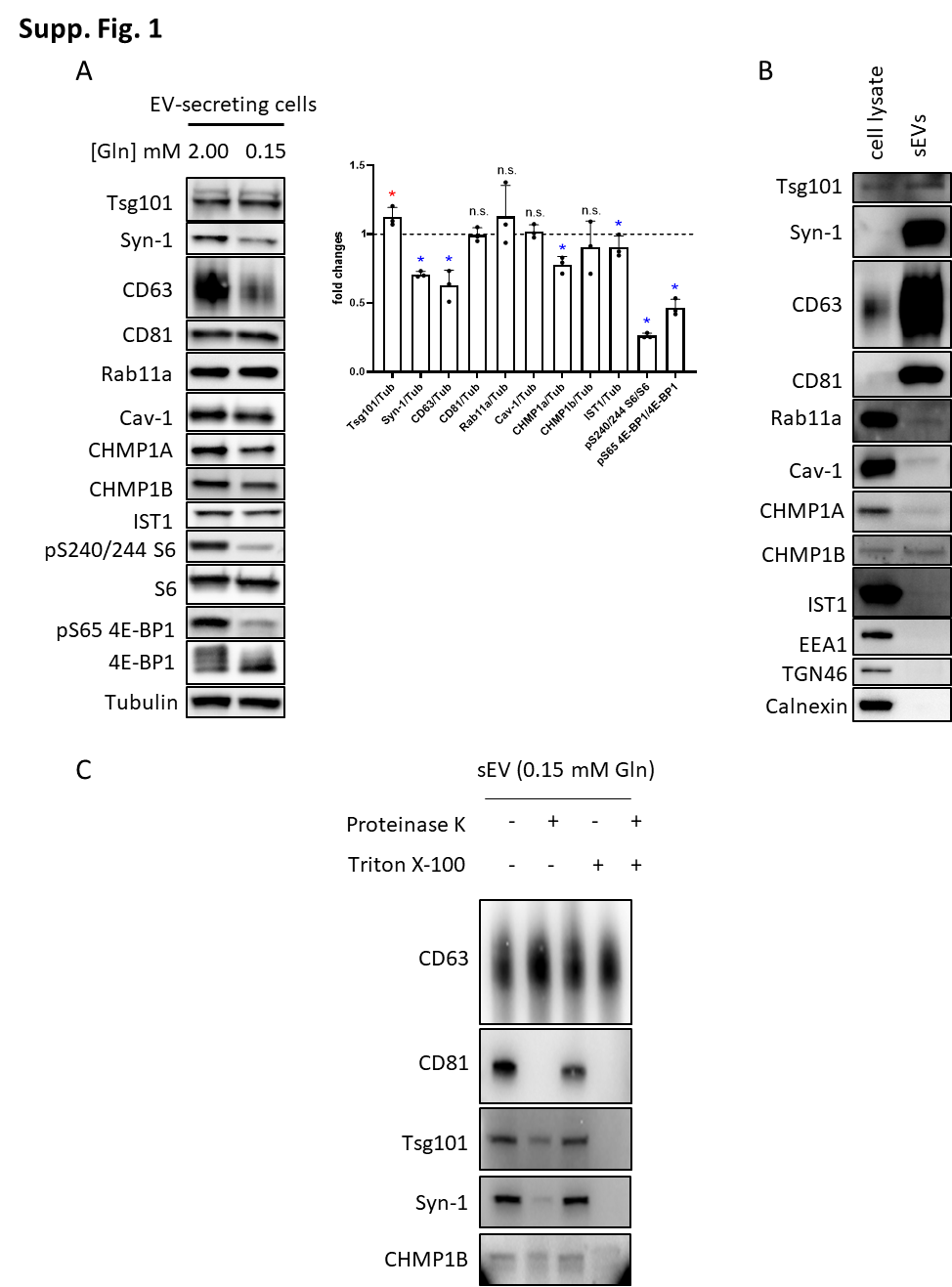


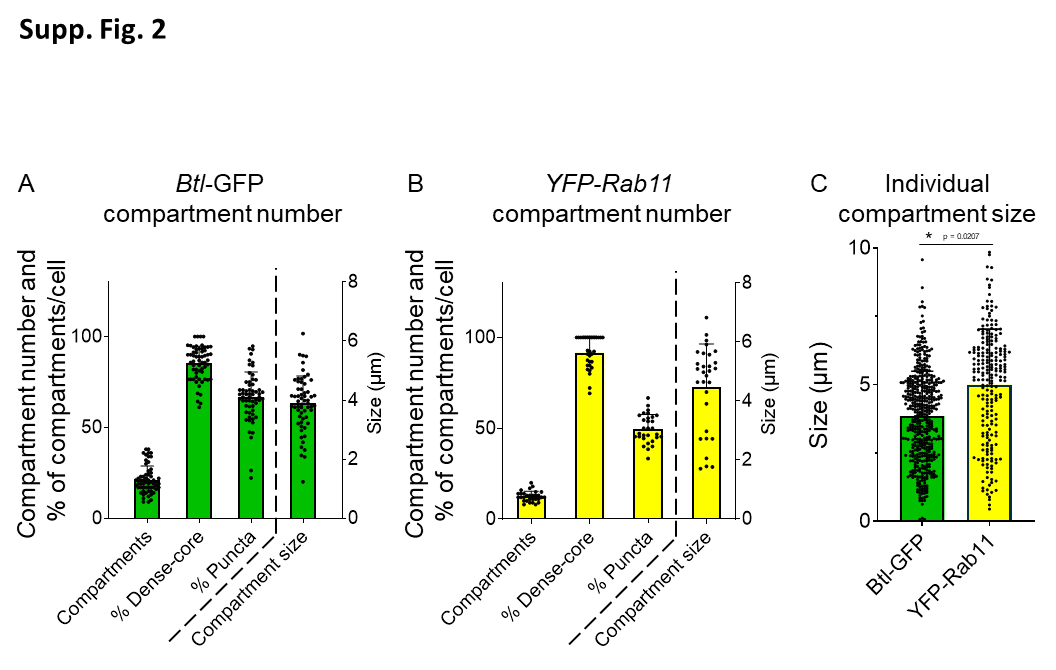


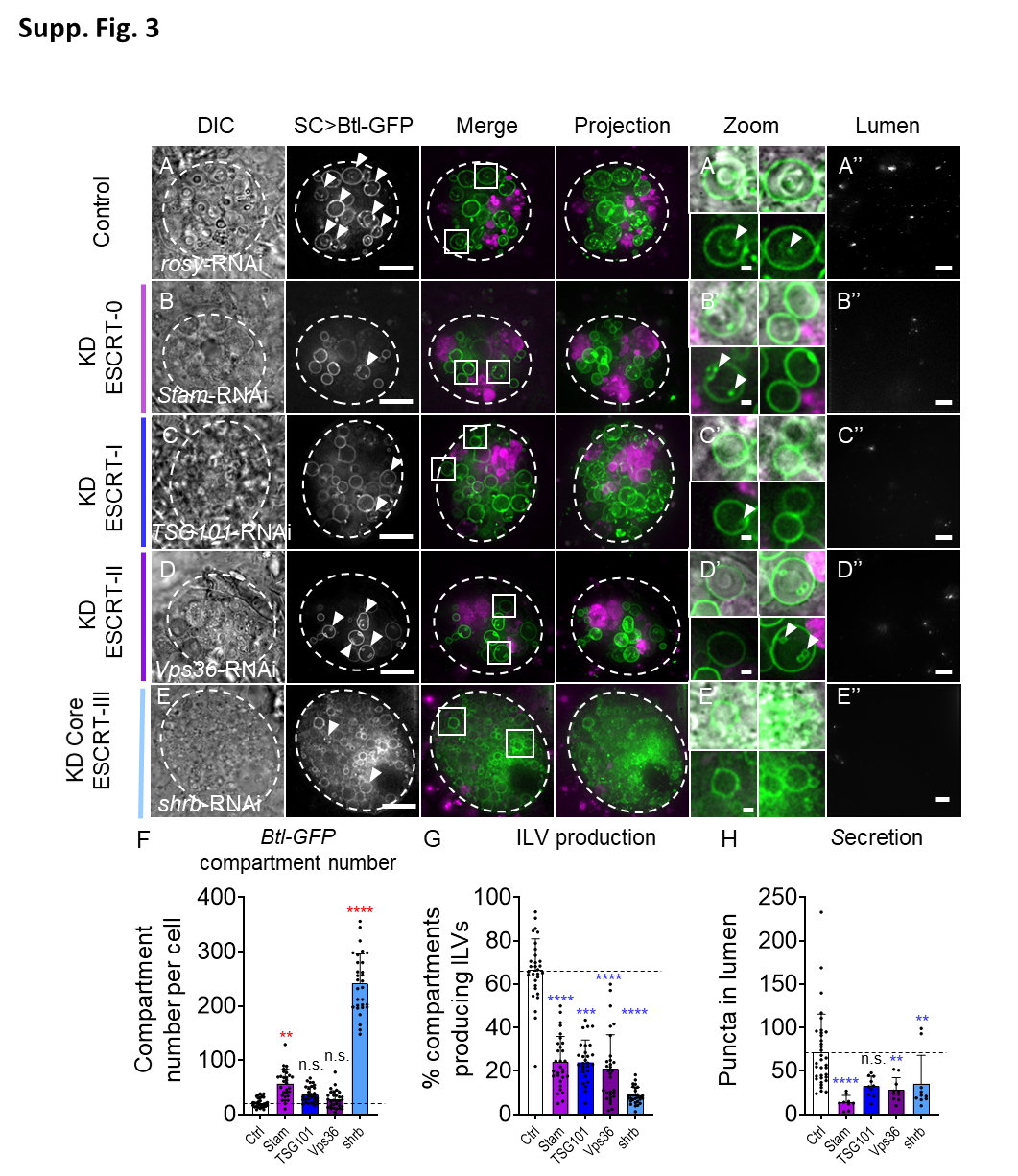


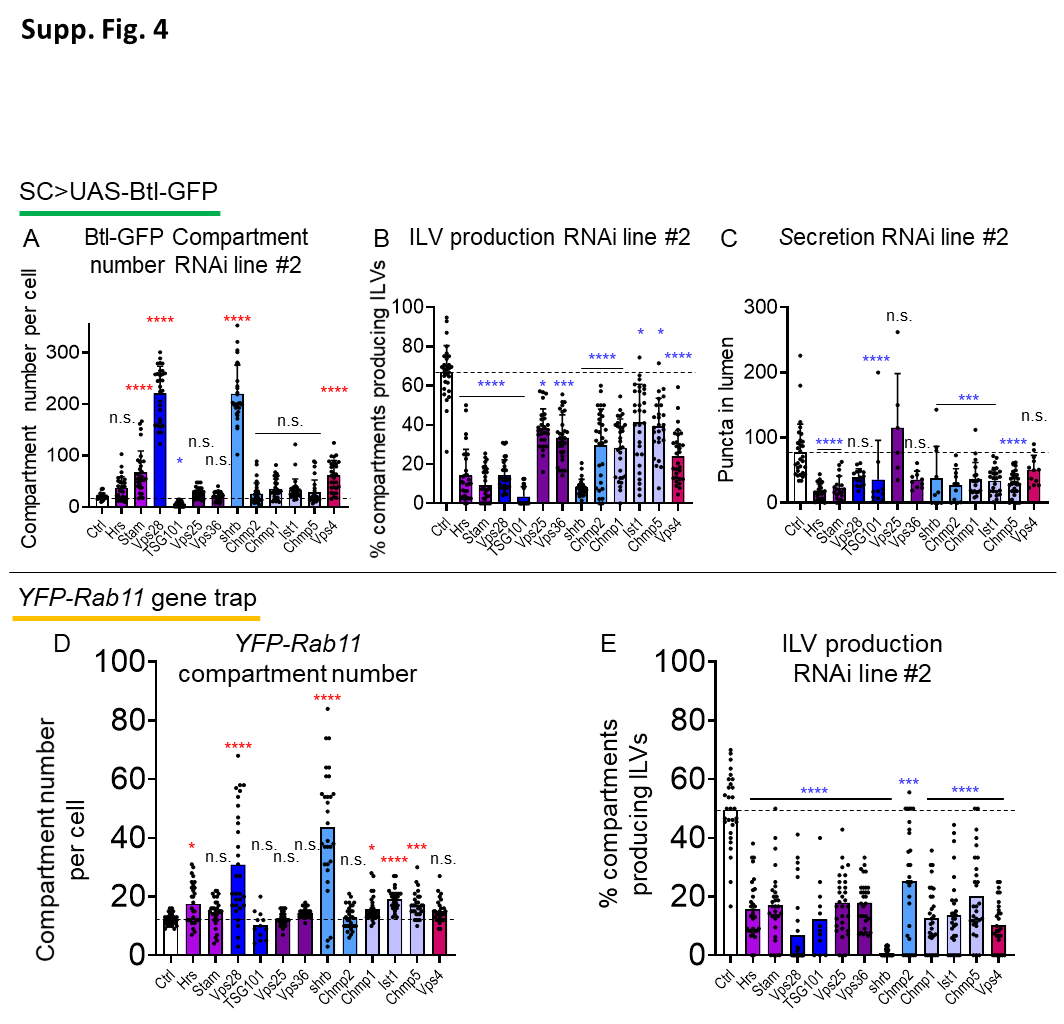


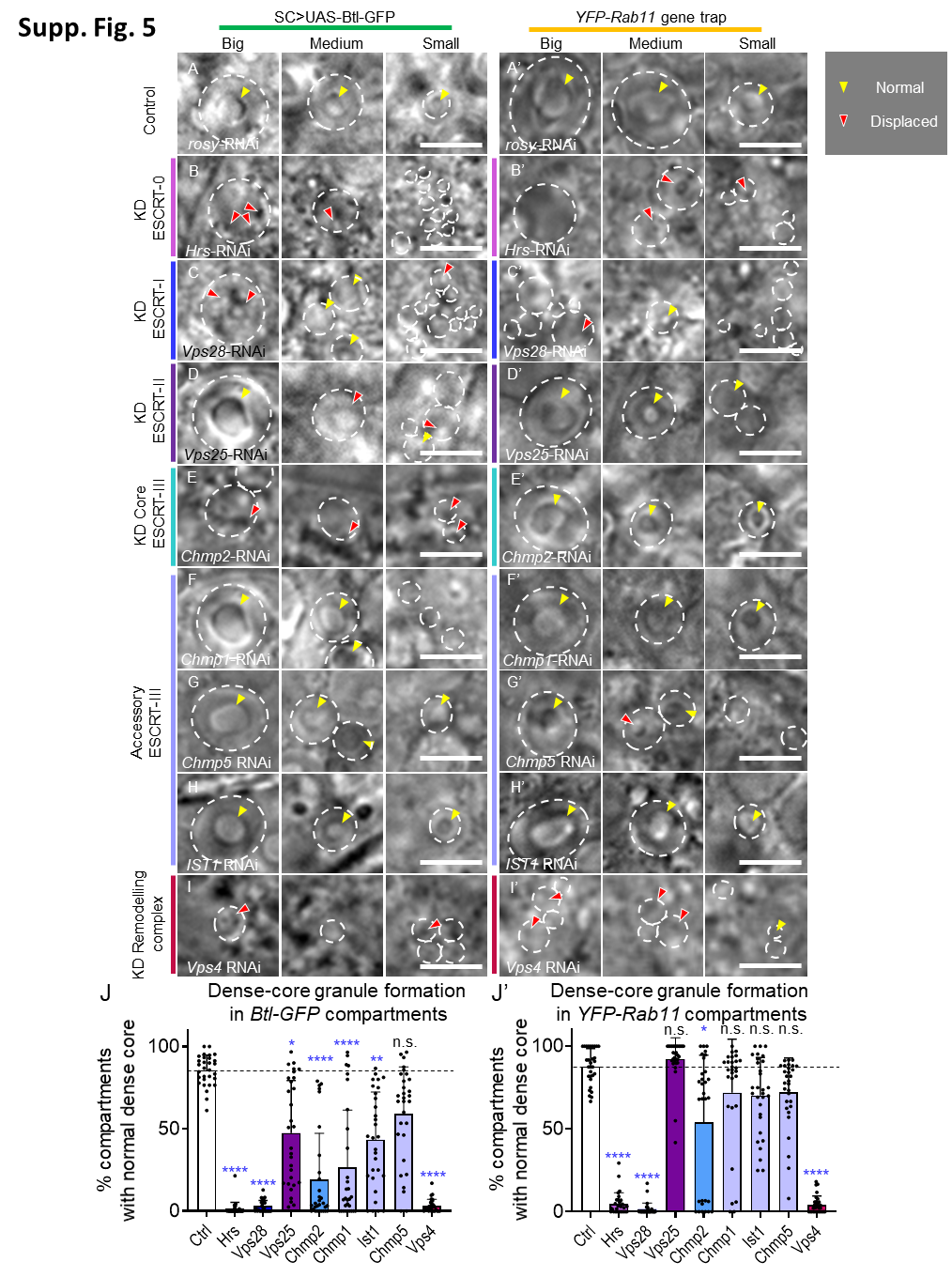


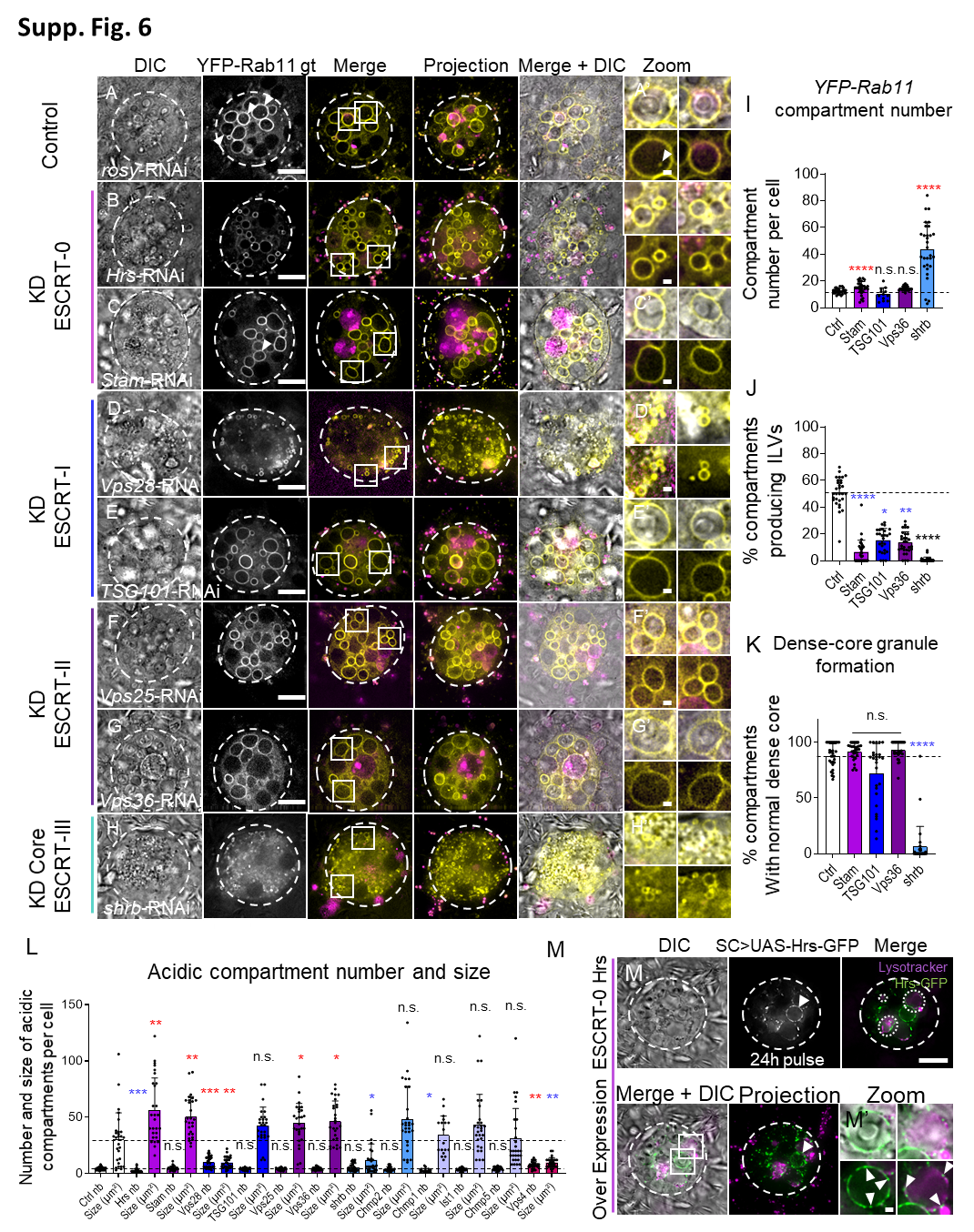


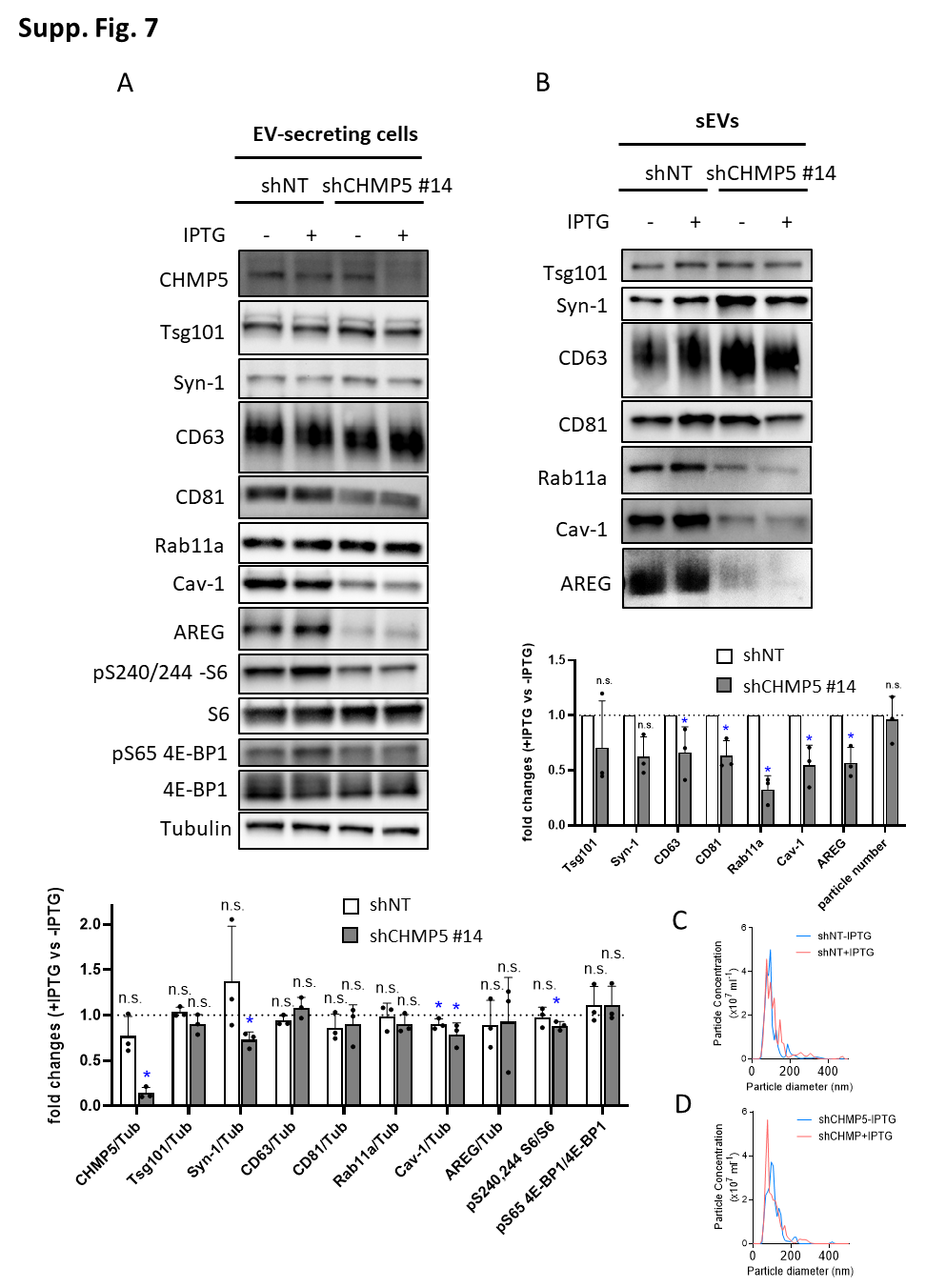


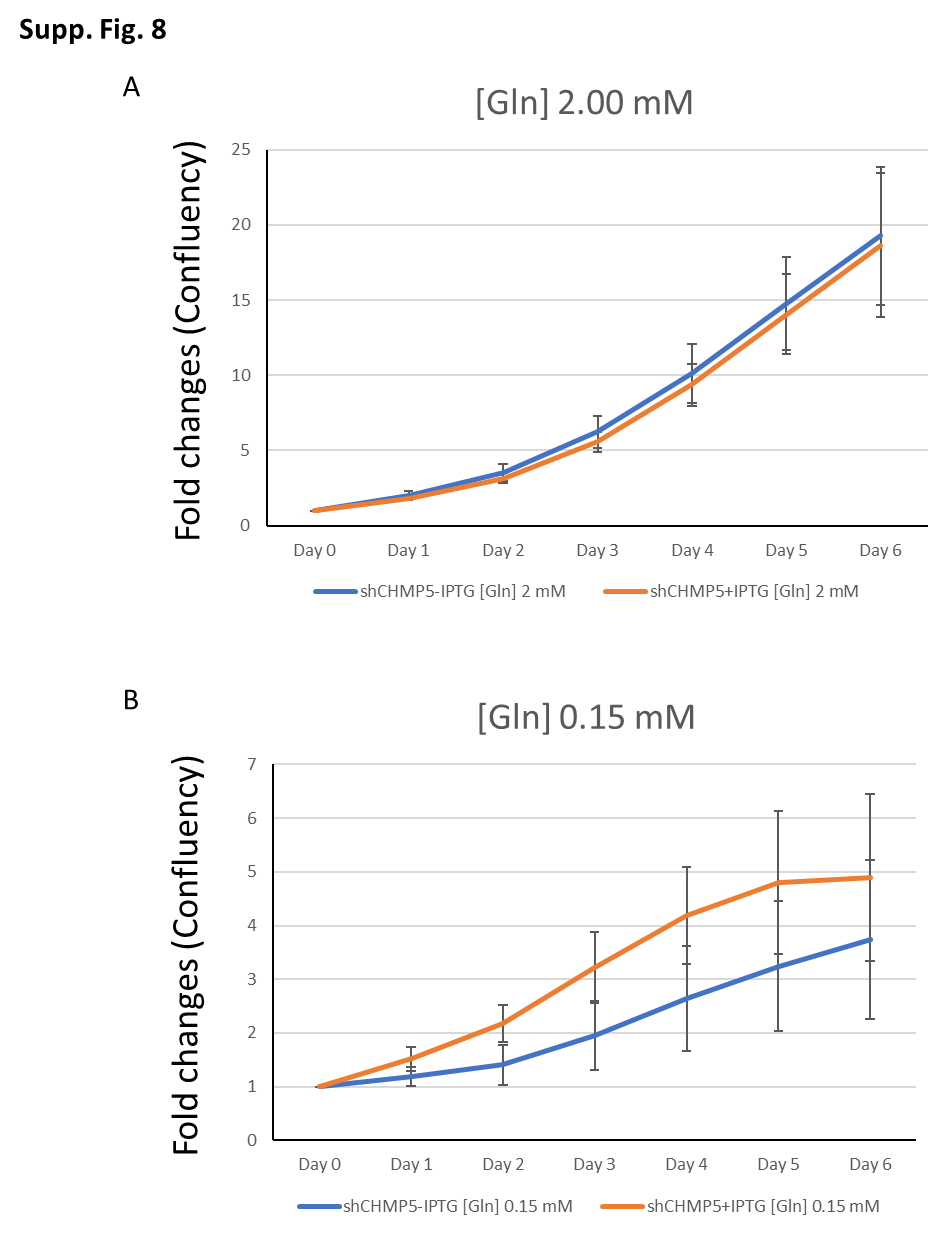

Supplement: Supplementary file 1 — Supporting Information [file JEV2-12-12311-s006.docx]
