# Supplementary figures and images for: Accessory ESCRT‐III proteins are conserved and selective regulators of Rab11a‐exosome formation
Source: J Extracell Vesicles. 2023 Mar 5;12(3):12311. doi: 10.1002/jev2.12311 (PMC9986085; doi:10.1002/jev2.12311)

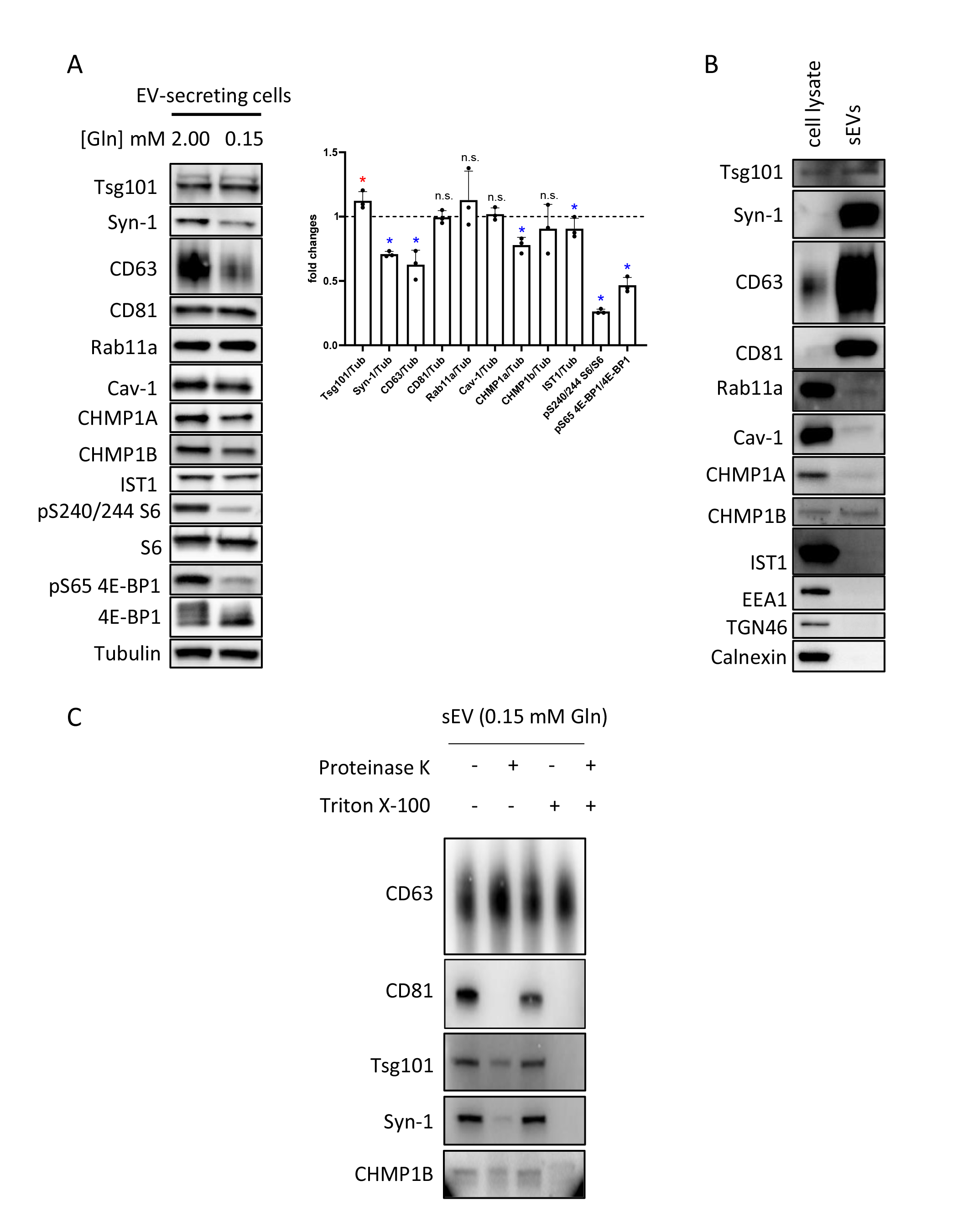

Supplement: Supplementary file 2 — Supporting Information [file JEV2-12-12311-s008.tif]

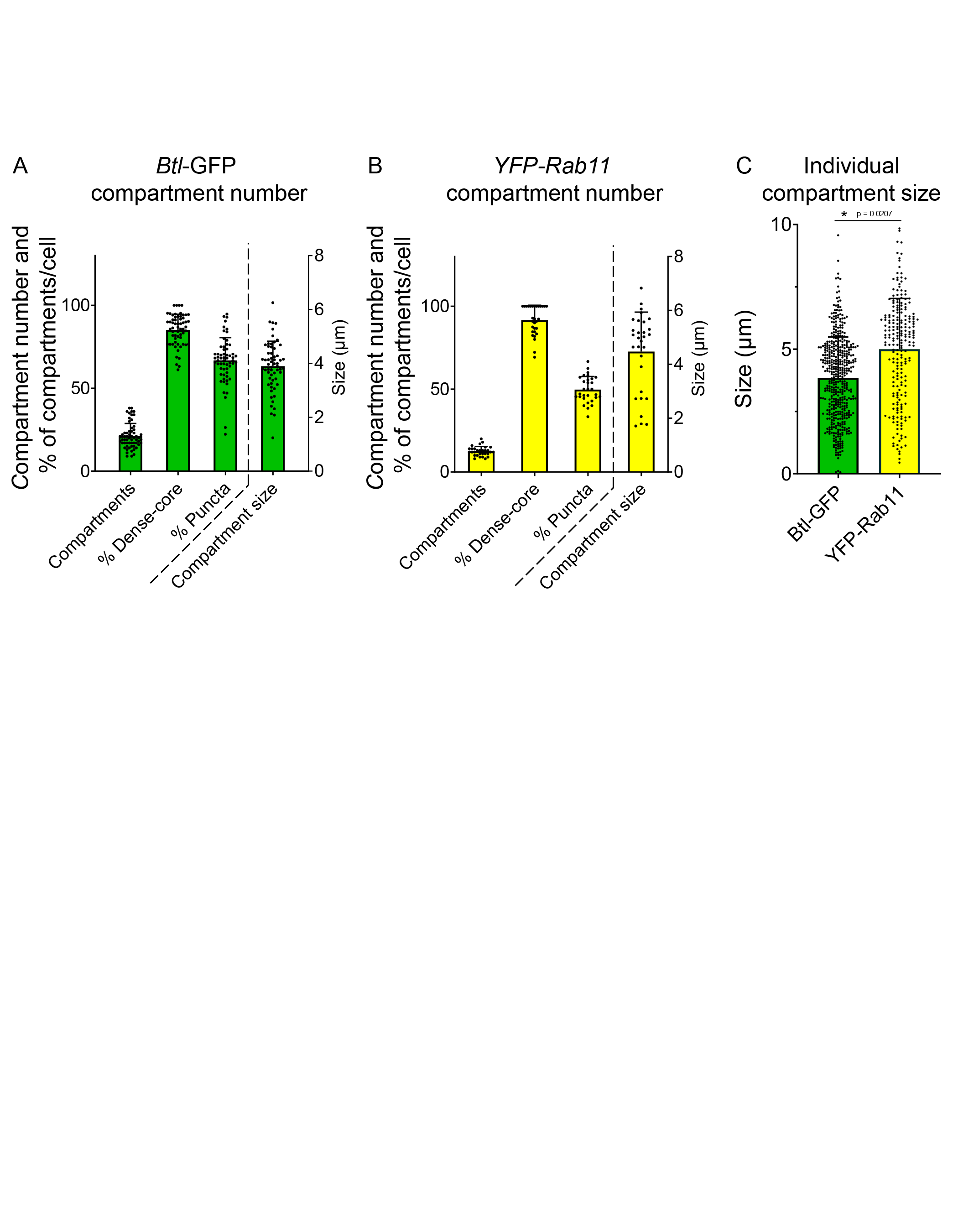

Supplement: Supplementary file 3 — Supporting Information [file JEV2-12-12311-s010.tif]

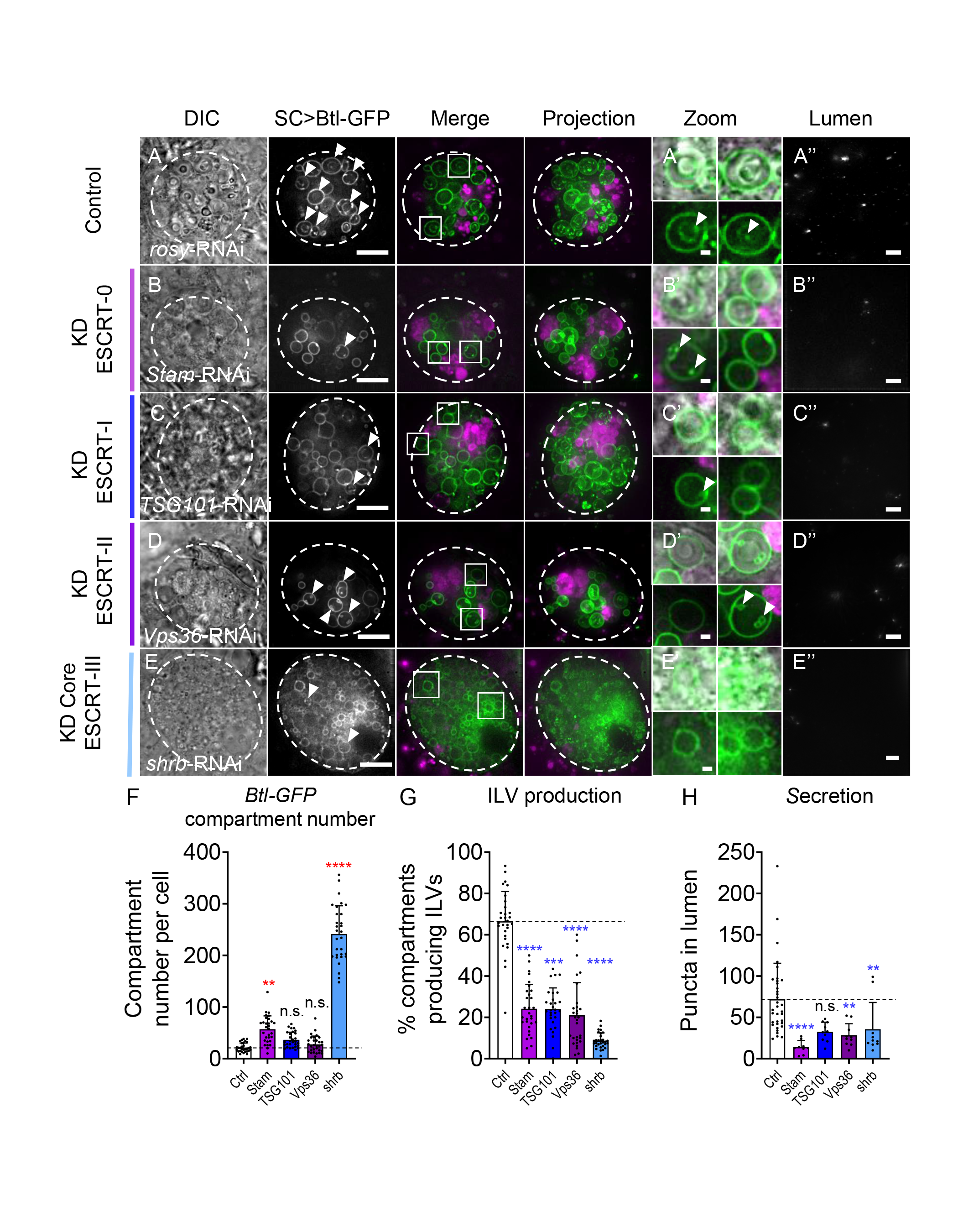

Supplement: Supplementary file 4 — Supporting Information [file JEV2-12-12311-s001.tif]

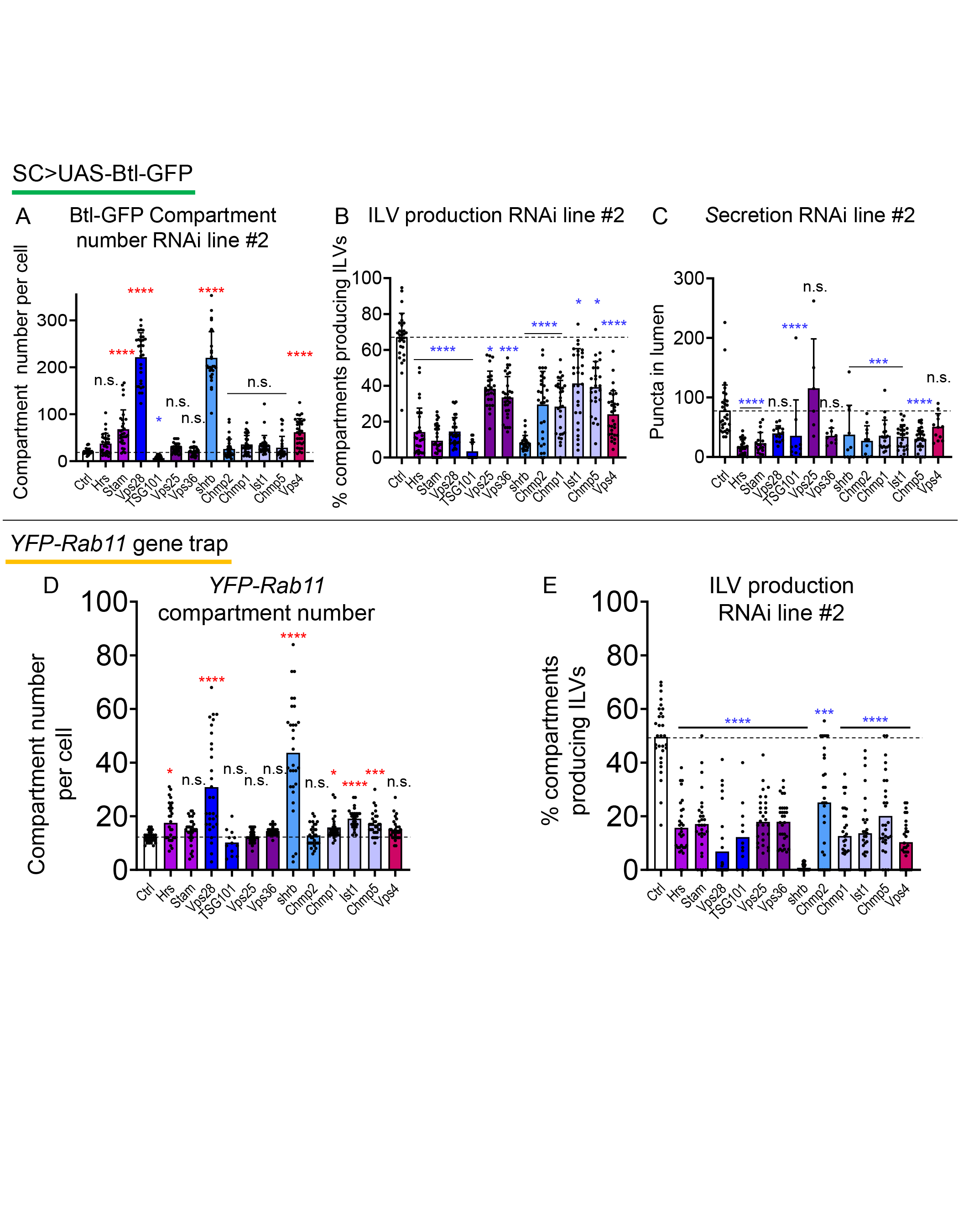

Supplement: Supplementary file 5 — Supporting Information [file JEV2-12-12311-s002.tif]

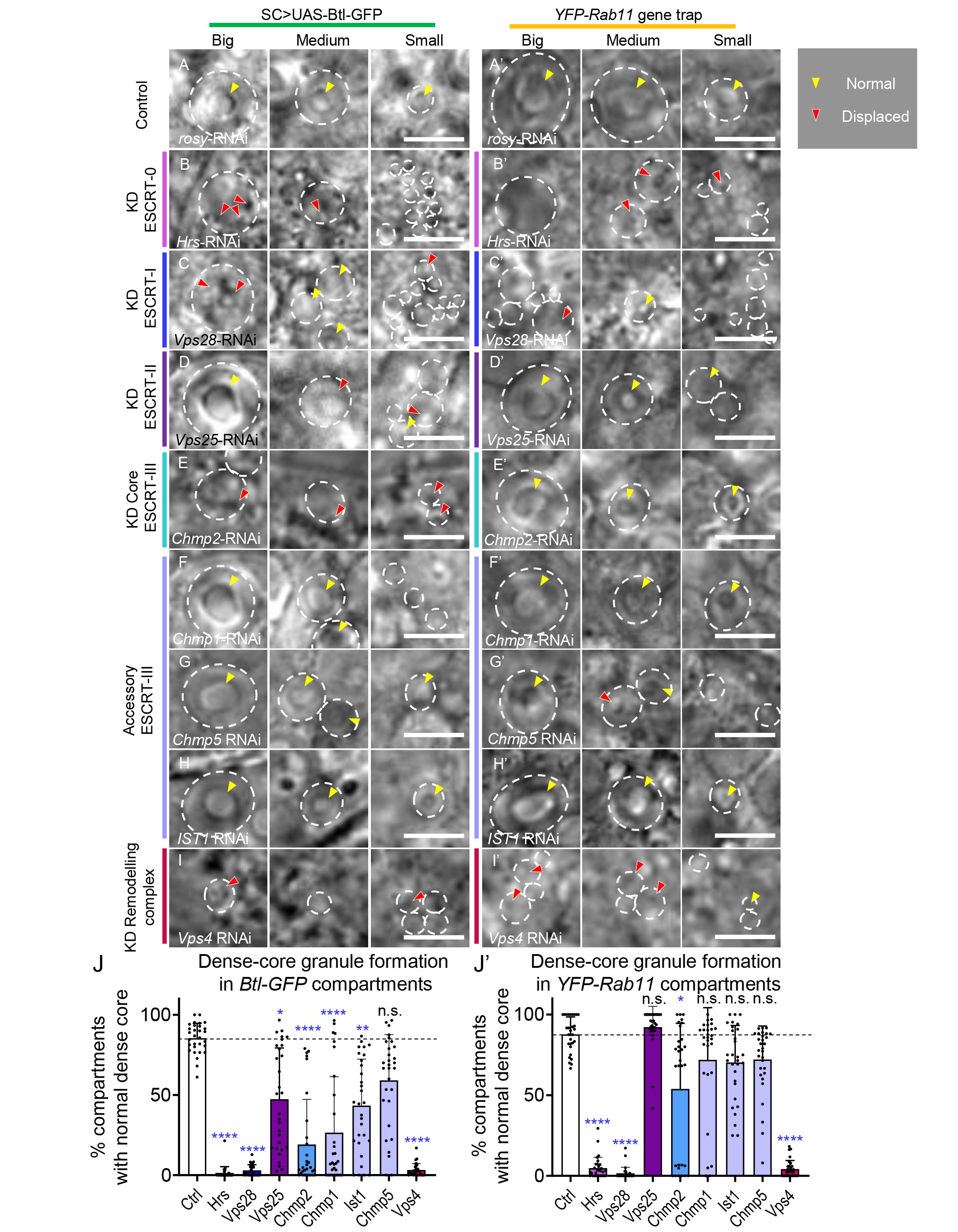

Supplement: Supplementary file 6 — Supporting Information [file JEV2-12-12311-s003.tif]

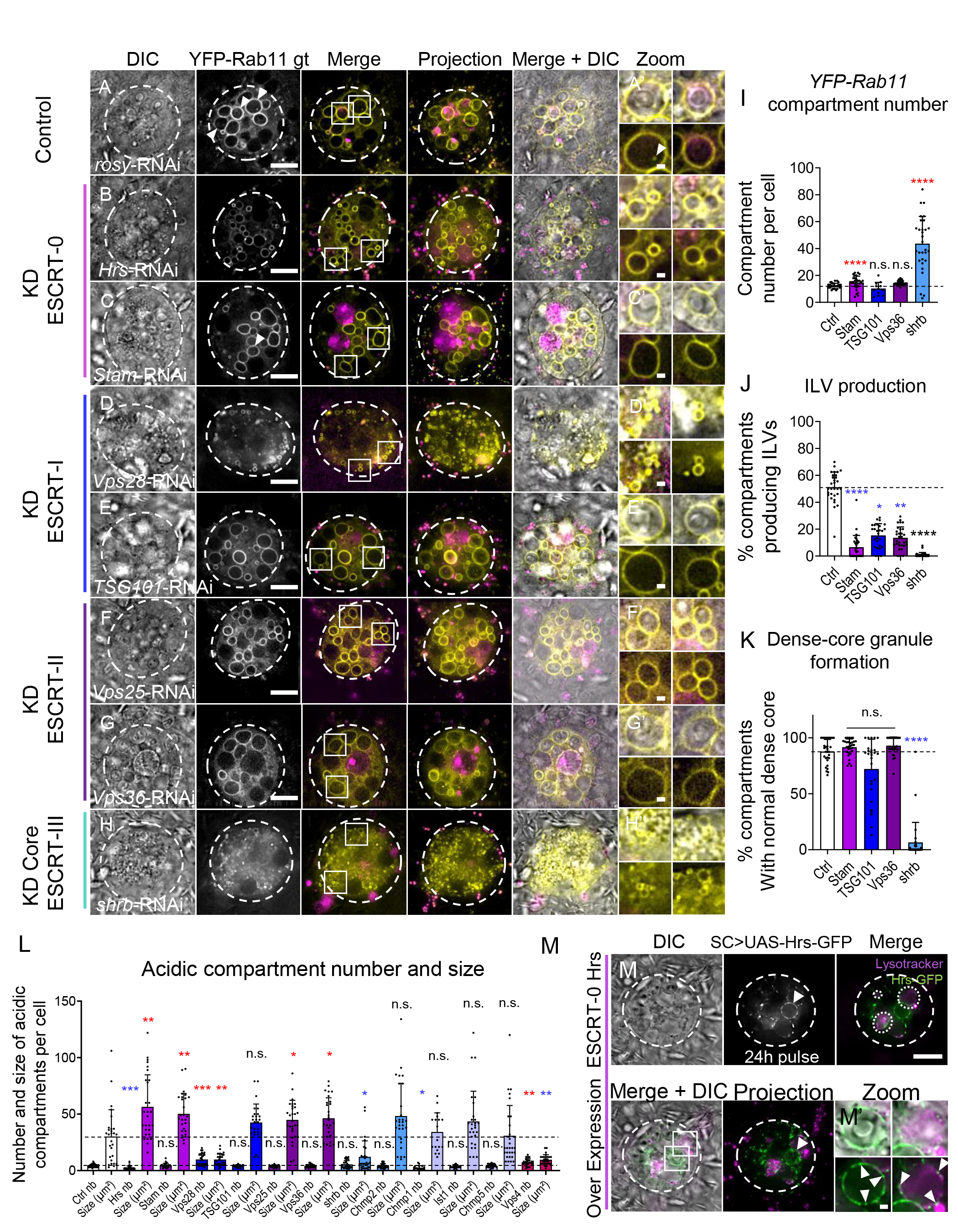

Supplement: Supplementary file 7 — Supporting Information [file JEV2-12-12311-s009.tif]

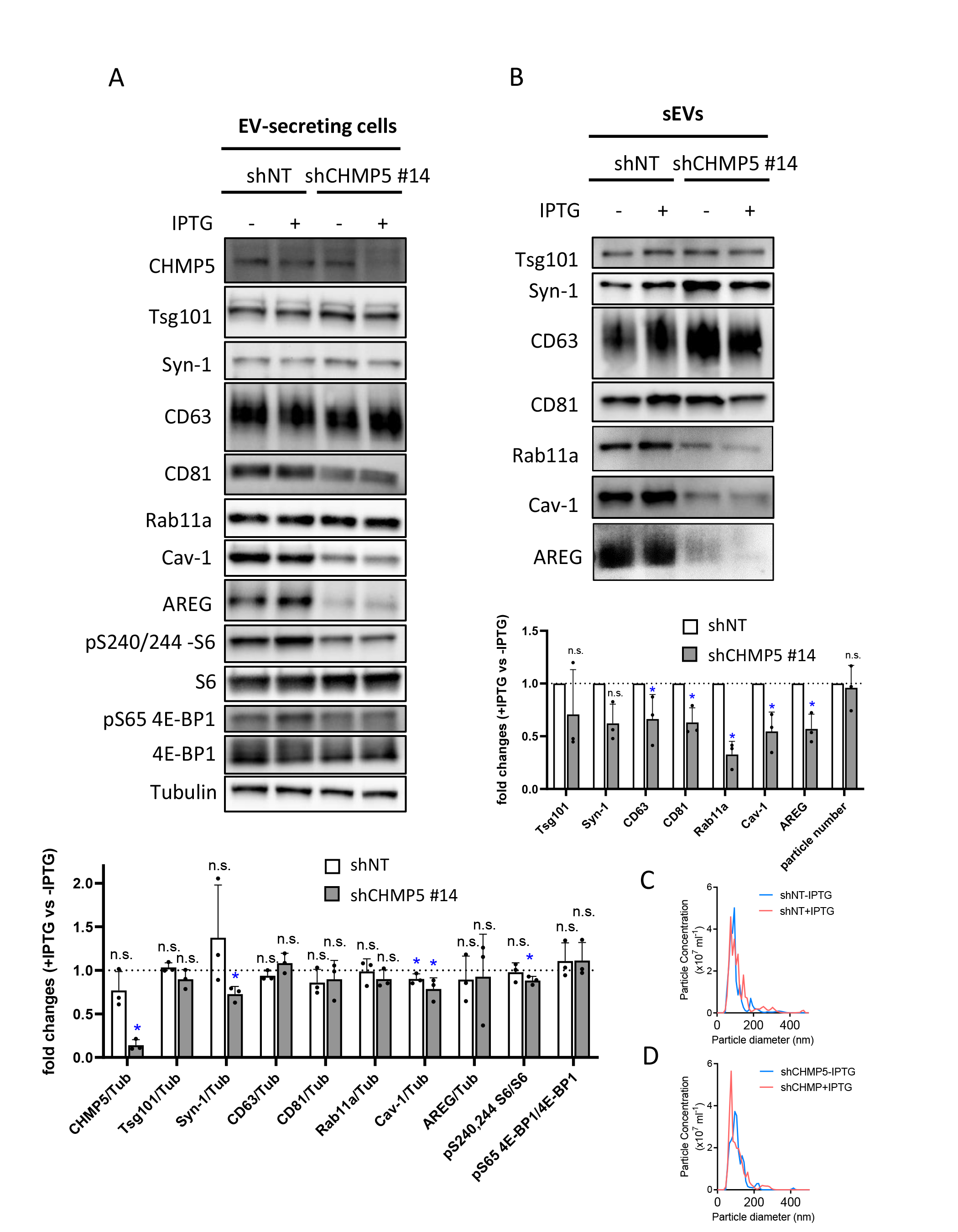

Supplement: Supplementary file 8 — Supporting Information [file JEV2-12-12311-s014.tif]

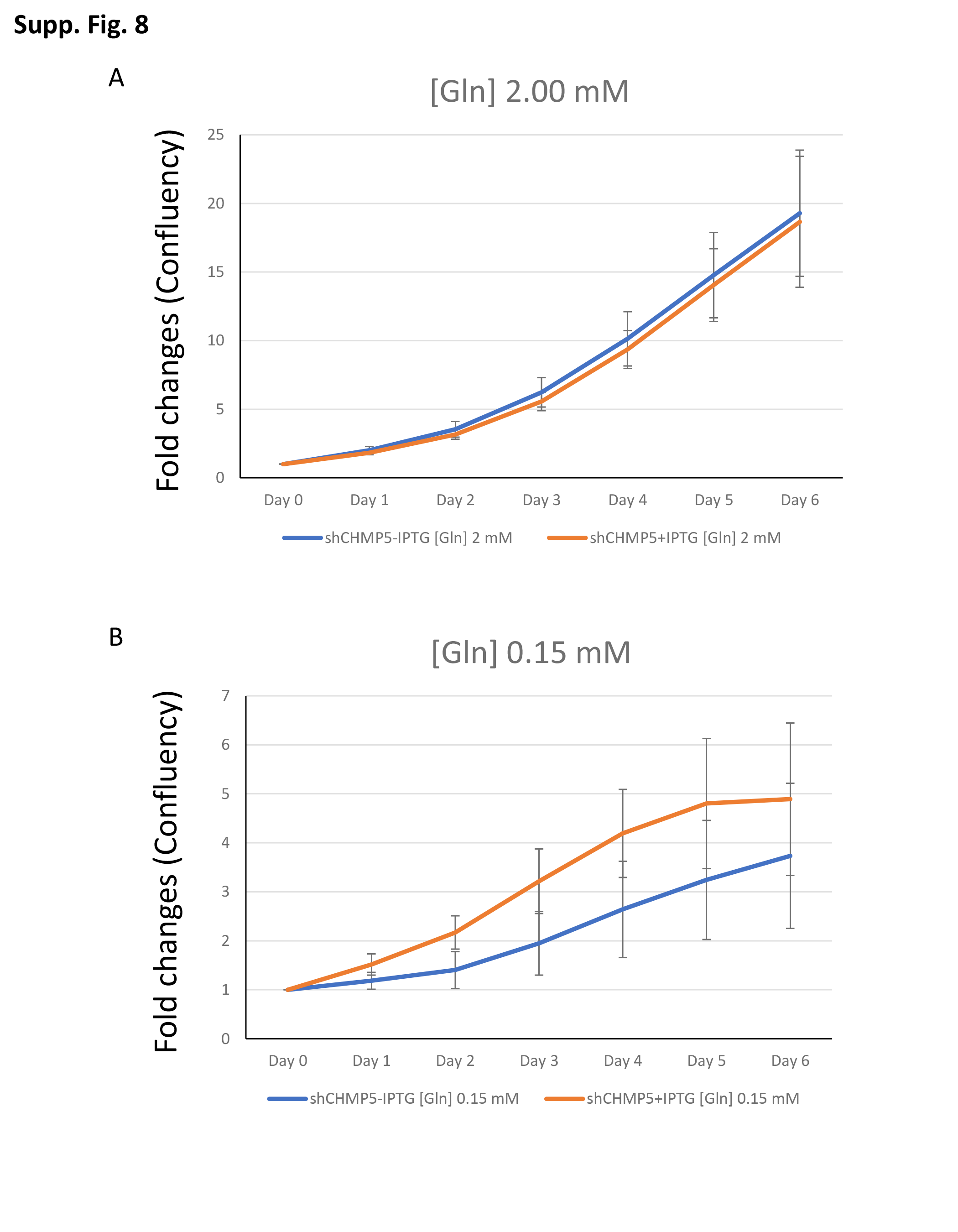

Supplement: Supplementary file 9 — Supporting Information [file JEV2-12-12311-s005.tif]
